# Unlocking the potential of sugarcane bagasse: a comprehensive analysis for advanced energy conversion

**DOI:** 10.1186/s40643-025-00878-5

**Published:** 2025-06-17

**Authors:** Nestor Proenza Pérez, Javier Alejandro Rodríguez Travieso, Elbis D´Espaux Shelton, Daniel Travieso Pedroso, Einara Blanco Machin, Celso Eduardo Tuna, José Luz Silveira

**Affiliations:** 1 School of Engineering at Guaratinguetá, Department of Chemistry and Energy, Laboratory of Optimization of Energetic Systems (LOSE) and Bioenergy Research Institute (IPBEN-UNESP), São Paulo State University, São Paulo, Brazil; 2 Faculty of Educational Technology, Department of Mathematics, University of Computer Sciences, Havana, Cuba; 3 Facultad de Ingeniería, Departamento de Ingeniería Mecánica, Universidad del Bío-Bío, Concepción, Chile; 4 Facultad de Ingeniería, Departamento de Ingeniería Mecánica, Universidad de Concepción, Concepción, Chile

**Keywords:** Sugarcane bagasse, Densities, Physical properties, Particle size, Models

## Abstract

The sugarcane bagasse was analyzed for Particle Size Distribution (PSD) with a mean geometric diameter of 0.722 mm. Various standard techniques assessed its physical and chemical properties, including density measurements, higher heating value (HHV), thermogravimetric analysis (TGA/DTA), and compositional, proximate, ultimate, and CHNS/O analysis. The raw bagasse showed higher volatile matter, fixed carbon, ash content, and HHV of 16 MJ/kg, with lower moisture content (8.71%). Thermal analysis indicated a peak degradation temperature for organic matter at 310–330 °C, and bagasse exhibited a higher combustion index than fossil fuels and other biomasses. Logarithmic models were obtained to determine the real, particle, and apparent densities of bagasse with the mean particle size within the 0.075–9.5 mm range, showing adequate results for particles with a mean diameter greater than 0.15 mm. For smaller particles, the reported errors were 12.6%, 8.23%, and 28%, respectively. These findings highlight sugarcane bagasse's significant potential for thermochemical conversion systems and its importance in selecting and designing fluidized bed technologies like pneumatic conveying, drying, combustion, and gasification equipment.

**Graphical Abstract:**

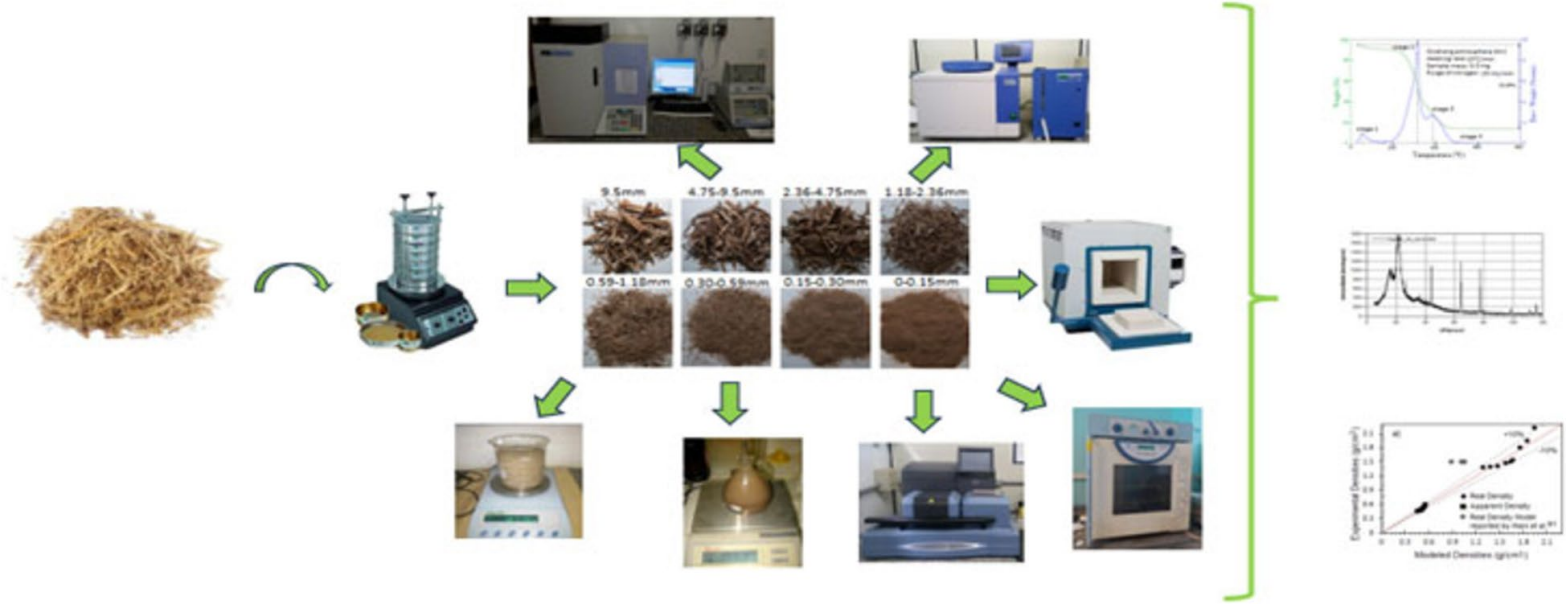

## Introduction

The significant development of the sugarcane industry in the great majority of countries producing it, such as Brazil, China, India, Thailand, and Australia, has led to a quick increase in production and industrial processing (Mohammadi et al. 2020). The sugarcane harvest in the producing countries generates approximately 1.9 Gt/year. The residues produced correspond to a global production of approximately 40 GW of electricity or 300 GWh/year, considering the eight major producing countries (Kabeyi and Olanrewaju 2023; Okure et al. 2006). 

In Brazil, the estimated total production of milled sugarcane during the harvest of 2022/23 was 610.1 million tons, with an increase of 5.4% compared to the harvest of 2021/22, which was 585.4 million tons, representing an increase of 24.7 million tons from the previous harvest (CONAB 2022, 2021). Nevertheless, despite that increase, Brazil is still considered the biggest sugarcane producer in the world, responsible for approximately 25% of the world’s production (Ogura et al. 2022).

The residue derived from the sugarcane milling process for extracting juice is called bagasse. It is not a homogeneous material since it comprises clusters of particles of different shapes and sizes. Around 30–35% of its structure is composed of small and slightly rounded particles, called "pith," which are derived from the core of the plant; the remaining 65–70% of its structure is composed of particles in the shape of parallelepipeds, coming from the "rind," and larger particles in the shape of cylindrical rods, commonly called fibers, which originate from the sugarcane, after it passes through the extraction process of the juice in the mills and alcohol distilleries (Mahmud and Anannya 2021; Wong Sak Hoi and Martincigh 2013). Characterization of sugarcane bagasse involves analyzing its physical, chemical, and thermal properties to understand its potential applications and the most suitable valorization methods. The principal organic components of sugarcane bagasse are a complex mixture of natural polymers of carbohydrates known as cellulose (30–50%), which is a polymer with a high molecular weight, composed primarily of glucose units; hemicellulose (20–30%), composed mainly of xylose units and small amounts of arabinose and lignin (15–25%), that is a complex substance composed mainly of aromatic phenolic compounds, which gives the sugarcane fiber its rigidity and hardness. In addition, many other species and other chemicals collectively called extractives are deposited in the plant cell wall, forming part of the composition and the ashes (Ungureanu et al. 2022). 

**Fig. 1:**
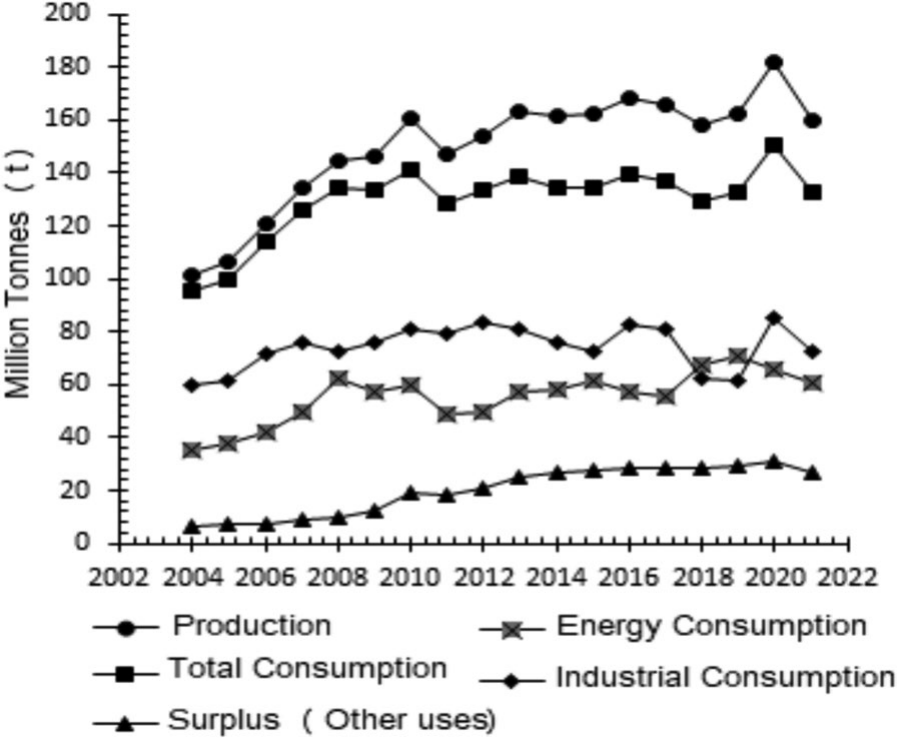
Brazil's production, consumption, and residue availability (EPE 2022)

The production, consumption, and availability of bagasse in Brazil are shown in Fig. 1. Usually, the bagasse is used in sugar-ethanol mills as fuel in the boilers to provide heat and electricity necessary for the manufacturing processes, which allows them to be energetically self-sufficient under certain conditions (cogeneration), which is not common in the case of other industries (Fioranelli and Bizzo 2023). In Fig. 1, energy consumption is referred to as the production of energy in sugar and alcohol mills, and industrial consumption refers to the use of bagasse in food and beverage elaboration and the paper and pulp industry. It is possible to observe an increase in the quantity of bagasse available (surplus) used for power generation (electricity) year after year, reflecting an increase in the supply of more than 50% from 2003/2004–2022/2023. This has been possible due to the increased interest, in recent years, of the sugar and alcohol mills that export surplus energy to the grid (Fioranelli and Bizzo 2023). In the current conditions of the sugar mills (season 2022/2023), this means producing around 153·10^6^ tons of sugarcane bagasse with 50% moisture (wet basis). One ton of sugarcane generated approximately 250–280 kg of bagasse, depending on whether it was processed on a wet or dry basis (Ogura et al. 2022).

From this point of view (Fig. 1), no bagasse is available for other uses. This is not entirely true because steam production is much higher than the need for sugarcane processing, even when using equipment with low conversion efficiency, so bagasse is often burned during the inter-season period to reduce the extensive stocks of large spaces, or even part of the bagasse is sold to other companies, especially to the orange juice industry, making it difficult to register it. For this reason, Brazilian Energy Balance (BEN) (EPE 2022) does not analyze if there is any bagasse residue for other purposes. 

However, according to Kabeyi and Olanrewaju (2023), a possible alternative to diversify the use of sugarcane bagasse would be the production of electricity on a large scale by introducing technological improvements to current conventional systems and the viability of proposed new systems. Fioranelli and Bizzo (2023) report that the replacement of traditional boilers for more powerful ones that can generate a much greater volume of steam with a much higher temperature associated with condensation-extraction cycles, known as CEST (*Condensing Extraction Steam Turbine*), which substantially improves the energy efficiency of bagasse burning and the amount of thermal energy that can be generated, allowing a higher production of electricity with approximately half the consumption of bagasse that there is nowadays and, therefore, a higher volume of a surplus of bagasse would be available. 

The Biomass Integrated Gasifier/Gas Turbine Combined Cycle (BIG-GTCC) represents an advanced technology (Fioranelli and Bizzo 2023; Machin et al. 2021) that shows potential to compete with CEST technology, resulting in increased electricity production capacity per unit of biomass processed. Given the characteristics of sugarcane bagasse, fluidized bed or entrained flow gasification technologies are deemed suitable for integration into BIG-GTCC systems.

A study conducted by Machin et al. in 2021 (Machin et al. 2021) presented an economic and environmental evaluation of bagasse gasification at a Brazilian sugar cane mill. The research examined the implementation of torrefaction technology as a wet pretreatment method for bagasse, aiming to address feeding issues in the gasifier's continuous operation when using this powdered raw material. The study utilized a life cycle assessment to determine the environmental impacts following the integration of BIG-GTCC technology. The results indicated that gasifying the excess bagasse from the plant is the most economical and environmentally favorable choice. 

The knowledge of the sugarcane bagasse's primary physical, chemical, and geometric characteristics is of great importance for its value as an energy source and for knowing the effects of composition on the chemical kinetics of the reactions and other parameters of the thermochemical conversion process. Regardless of the process, knowledge of bagasse properties is necessary to improve the process, design, and selection of equipment involved in the different thermochemical conversion facilities. However, the bagasse characterization will depend on factors such as plant species, season, type of harvest (manual or mechanized), soil type, juice extraction process, fertilizer type, and others (Lenço et al. 2020).

**Fig. 2:**
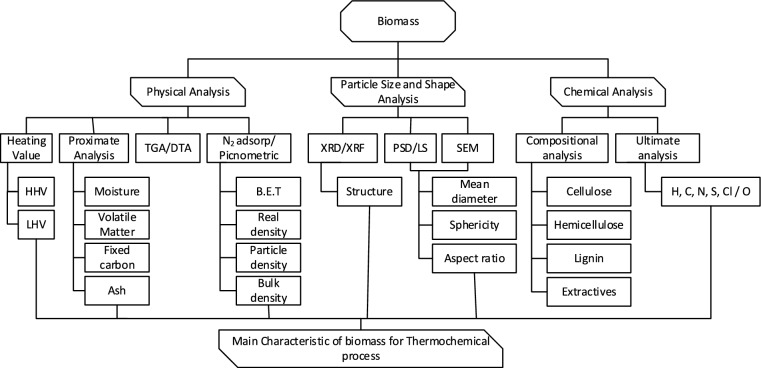
Routes of characterization of biomass for thermochemical conversion

Some different techniques and tools enable more comprehensive and accurate characterization of biomass materials; some of the most important for thermochemical conversion processes are shown in Fig. 2. Instrumental methods used for this purpose include X-ray diffraction spectrometry (XRD), X-ray fluorescence (XRF), Light scattering (LS), and scanning electron microscopy (SEM). Thermogravimetric (TGA) and Differential Thermal Analysis (DTA) are used as thermal analysis techniques, while gas adsorption and gaseous psychometrics provide particles’ physical information (Jiang et al. 2019).

Numerous studies applying some of these techniques can be found in specialized literature; however, information is usually segregated into different studies for particular purposes for the same type of biomass. Combining all available information is essential to comprehensively understand the phenomena in energy conversion systems. The properties of the biomass and the specific characteristics of the conversion process are closely linked and depend on each other. Thus, the choice of the most suitable type of biomass is influenced by the specific characteristics of the thermochemical conversion process that will be used. At the same time, the properties of the selected biomass type are essential. They will directly influence the choice of one type of conversion technology, depending on the difficulties that may arise with the treatment of biomass (Alves et al. 2023).

The main properties of the biomasses considered necessary in the processes of thermal conversion such as combustion, gasification, and pyrolysis are density, moisture content, chemical composition, physical analysis, higher and lower heating value, fixed carbon/volatile material ratio, cellulose/lignin ratio, sphericity, aspect ratio, among others (Toscano Miranda et al. 2021).

Biomass combustion is a chemical reaction involving a combustible material and an oxidizer (usually oxygen), releasing energy in the form of heat, and is the most widely used thermochemical process in industry for generating electricity and heat. Pyrolysis is a thermal decomposition process that occurs between 250 and 600 °C temperature under inert conditions, i.e. with little or no oxygen, which allows biomass to be converted into three fundamental fractions: pyrolytic liquid, biochar and biogas, which can be used to produce energy, and in the production of chemical industry by-products, and gasification is the thermal conversion of biomass into a gaseous mixture (fuel) in the presence of an oxidizing agent under sub-stoichiometric conditions. The main compounds formed in this process are carbon monoxide (CO), carbon dioxide (CO_2_), hydrogen (H_2_), methane (CH_4_), and nitrogen (N_2_), with traces of hydrocarbons, as well as tar and particulates. The oxidizing agents can be air, pure oxygen, or steam (Clemente-Castro et al. 2023).

The detailed analysis of these parameters allows the determination of viability and type of thermochemical conversion technology that should be used in each case. Thus, physicochemical and geometrical characterization of biomass and their ashes allow us to obtain information on how their thermal decomposition will be, foreseeing the rate of devolatilization, as well as possible problems of sintering and melting of ashes, which can cause severe problems of fouling of heat exchange surfaces and reactor walls. Density and heating value can influence logistical considerations and transport and storage costs. While geometrical characteristics and particle size distribution are closely related to fluid-dynamics behavior in fluidized bed technologies, being able to directly affects the performance and quality of the products obtained (Balag et al. 2023).

Several researchers have conducted several studies involving sugarcane bagasse, mainly focused on the pretreatment of bagasse to separate its main components for bioethanol production (Jin et al. 2020; Shukla et al. 2023). Andrade and Colodette (2014) characterize the fundamental composition, the fibers, and the pith, evaluating their influence in the production of dissolving pulp grids. Another study reported by Szczerbowski et al. (2014), performs a comparative study of the composition of carbohydrate and non-carbohydrate components of bagasse and sugarcane straw of São Paulo state in Brazil to compare the source of fermentable sugars for bioprocesses. Mixtures of different types of bagasse are characterized by Rocha et al. (Rocha et al. 2015), from the physicochemical point of view. The study is primarily focused on determining the cellulose, hemicellulose, and lignin contents, as well as the amount of extractives and ash of sixty samples of different types of bagasse to verify the possible differences regarding their elemental composition and the relation between different components. Other studies focus on the fluid dynamics behavior of bagasse particles, or mixtures of this biomass with other materials, evaluating the tendency of mixing and segregation, in which the knowledge of the physical and geometric parameters of the particles involved is essential (Lenço et al. 2020; Ramirez-Quintero and Bizzo 2018). Recently, a study was conducted by Najafi et al. using various sugarcane residues like sugarcane straw, sugarcane bagasse, and sugarcane bagasse pith, performing a comparative evaluation of the main physicochemical properties of these residues for thermal conversion processes (Najafi et al. 2023).

Developing novel thermochemical conversion systems like fluidized bed gasification systems for sugarcane bagasse, which can have a stable operation in ongoing and variable load conditions, is an important task today. The future is even more promising, considering its application in BIG/GTCC systems for electricity production (Machin et al. 2021). For this reason, the objective of this paper is precisely to determine the main physicochemical properties of the sugarcane bagasse produced in the state of São Paulo in function of the mean particle size distribution, with an emphasis on their use in thermochemical conversion systems, where it is essential to know these properties for a good selection, design, and construction of this type of equipment. Unlike other works reported before, this paper only focuses on sugarcane bagasse residue, but in a much more detailed way, since it does not only consider bagasse in its natural form but makes a study based on the different particle mean diameters obtained of particle size distribution, comparing its main physicochemical properties, including the densities. It is known that one of the main problems reported in fluidized bed systems is the fuel feeding system, when the biomass used is of the polydisperse type with a low density and high cohesiveness, as is the case of bagasse, being the equipment that generates the most difficulties in practice, hindering, in some cases, the investigations and even the deactivation of the systems (Sanchez and Lora 1994; Gómez et al. 1995). In the case of the need to adapt the particle size that will be fed to the fluidized bed system through conventional systems, the results presented in this work may assist in decision-making.

## Materials and methods

Approximately 150 kg of sugarcane bagasse was collected from the Serra mill in the central region of São Paulo (−22.012436, −48.000392). The bagasse was gathered from stored stacks located in the mill's backyard. The samples were a mixture of various sugarcane varieties from different geographical locations, soil types, harvest times, weather conditions, and harvest forms (manual or mechanized). These samples were collected from both the end and center of the stacks and then placed in 100-L plastic bags. After thorough mixing and division, one-quarter of the sample was tightly sealed in a plastic bag for laboratory analysis. The remaining bagasse was carefully stored in strong plastic bags and sealed for transportation.

In this study, the bagasse produced at the exit of the last mill is referred to as "bagasse in nature" or raw bagasse, consisting of a diverse range of polydisperse particles in varying sizes and shapes.

This particular milling tandem process, involving the imbibition process, is the most common and traditionally utilized in over 90% of the existing sugar and alcohol mills in Brazil (Lenço et al. 2020). The research includes several sugarcane varieties such as SP79-1049, SP91-1049, RB80-3280, RB867515, SP81-3250, RB855453, RB72454, and SP83-2847, which are widely used in Brazil, particularly in the central south region of São Paulo and the Brazilian Northeastern region (Oliveira et al. 2021).

### Geometrical analysis

#### Granulometric analysis

To determine the particle size distribution (PSD) of sugarcane bagasse and, subsequently, the geometric mean diameter, in this research, the method of mechanical standard sieving has been chosen, according to the recommendations of Castells et al. (2023). The standard procedure followed for the sieving analysis is the norm ASTM E828 (Standard Test Method for Designating the Size of RDF-3 From its Sieve Analysis) (A. E828-81,2004). Standard Tyler sieves have been used in series with the opening characteristics listed in Table 1: metal sieves with circular sections of metal mesh and square holes.

**Table 1:** Series of sieves used in the granulometric analysis of raw sugarcane bagasse

Sieves	Aperture size (mm)	Tyler Series
1	9.5	3/8”
2	4.75	4
3	2.36	8
4	1.18	14
5	0.59	28
6	0.3	48
7	0.15	100
8	–	bottom

For determining the mean diameter of a particle between two consecutive sieves (*d*_*pi*_), the expression reported by Gomez et al. (2012) has been used, known as a characteristic dimension of the particle.

1 $$d_{p,i} = \left[ {\frac{{\left( {X_{i}^{2} + X_{i + 1}^{2} } \right)\, \cdot \,\left( {X_{i} + X_{i + 1} } \right)}}{4}} \right]^{0,33}$$

*X*_*i*_ is the standardized opening of the sieve that allows the passage of the fraction (mm), and *X*_*i*+*1*_ is the standardized opening where the fraction is retained (mm).

The determination of the geometric mean diameter of Sauter, according to Kunni and Levespiel (1991), is calculated as follows:

2 $$\overline{d}_{p} = \left[ {\frac{1}{{\mathop \sum \nolimits_{i = 1}^{n} \left( {\frac{{x_{i} }}{{d_{p,i} }}} \right)}}} \right]$$

where *x*_*i*_ is the mass fraction of particles of size with a mean diameter equal to *d*_*pi*_, and* d*_*pi*_ is the arithmetic average of the opening between two adjacent sieves where the particles are retained (mm).

#### Procedure

The granulometric analysis was performed on 80 g of raw bagasse, placed in the upper sieve (sieve with the largest opening). Typical sieving equipment involves a nested column of sieves closed by a tamp. The conjunct has been attached to the sieving shaker Produtest, T Model from Brazil. Some previous experiments with this type of biomass have made it possible to prove that the proper sieving time, which is technically reliable and economical, is approximately 20 min (Gómez et al. 2012). That time must be the smallest amount in which the particle size distribution does not change significantly. After the shaking time was complete, the screens were removed from the equipment. The mass fraction retained on each sieve was carefully placed in a container and subsequently weighed in a digital electronic scale DIGIPESO brand DP-3000, with a precision of 0.01 g. These steps were repeated five times, following the criteria that the mass of the original sample of the test did not change by more than 5% from the previous mass value on that sieve.

#### Geometric analysis

The geometric characterization of sugarcane bagasse particles was done following the methodology reported by Pérez et al. (2019) to measure the main dimensions of bagasse particles. In this case, three different geometry models of the particles were assumed in relation to their main dimensions (length/width) due to the polydispersity character of the bagasse particles with a very heterogeneous distribution of sizes and shapes. Thus, particles in the form of fibers were modeled as cylinders, fine particles with a high length/width ratio as parallelepipeds, and small particles, pith type, were approximated to spherical models.

To determine the dimensions of the coarse particles, an electronic digital caliper, and an electronic digital thickness gauge were employed, with a resolution of 0.01 mm. Zeiss brand pith-type microscopes were utilized for smaller particles, including the model Stemi 2000 and an optical transmission microscope with bright-field illumination and semi-achromatic lenses (2λ, 2λ) of 2.5X, 5.0X, and 20X magnification. ImageJ software was utilized to analyze and process the images, enabling the measurement of primary dimensions and other significant geometrical properties, such as sphericity. The morphology of the biomass samples was analyzed using Scanning Electronic Microscope Equipment (SEM), QUORUM brand, and the Q150R ES model. The samples were spray-coated with gold particles to determine their morphological characteristics better. For the evaluation of the samples, 55- or 500-times magnifications were selected, depending on the size. The paper was elaborated separately because of the analysis's complexity, importance, and extension, and the main results and discussions are shown (Pérez et al. 2019).

### Chemical analysis

#### Ultimate analysis

The ultimate analysis was conducted to determine the content of five major elements in the organic phase: carbon (C), hydrogen (H), nitrogen (N), and sulfur (S). The analysis followed the ASTM D3176-3179 method using an Element Analyzer (Perkin-Elmer Series II, CHNS/O Analyzer 2400). All measurements were replicated three times, and the average value, adjusted for moisture content, is presented. The oxygen content was calculated by subtracting the combined percentages of C, H, N, S, and ash from 100%. The results are reported on a dry ash-free basis (daf).

#### Compositional analysis

The compositional analysis aims to determine the cellulose, lignin, hemicellulose, and extractives present in the biomass. These parameters were not determined experimentally in this study, as they were obtained from previously reported scientific publications that used the same type of biomass collected at the same region that the sample analyzed in this work. Two different methods are analyzed. The first method, sugarcane bagasse, is pretreated by steam explosion and alkaline delignification and then analyzed using a methodology adopted by Rocha et al. (2011), where the acid-insoluble lignin is determined using a modification of the Klason method. The methodology consists of two stages of acid hydrolysis of extractive-free material and the subsequent chromatographic quantification of sugars and degradation products contained in the hydrolysate and determination of acid-insoluble lignin by gravimetric analysis. The bagasse sample used in the analysis comprised particles with diameters between 0.25 and 1.19 mm. The techniques, equipment, and conversion factors used to obtain the components' concentrations converted into cellulose, lignin, hemicellulose, and extractives are described in detail in Rocha et al. (2011) (Rocha et al. 2012). The second method does not perform any pretreatment in the biomass. The extractive content was performed according to TAPPI 264 cm-97 and TAPPI T280 pm-99 (2006) standard procedures, respectively, and lignin levels according to TAPPI UM 250 and TAPPI 222 om-97 (T. Om-11,2011). Analysis for cellulose and hemicellulose content was performed by hydrolysis with sulfuric acid (TAPPI T204 cm-97) (T. Cm-07,2007), with subsequent analysis of total carbohydrate content by HPLC, according to researchers reported by Rocha et al. (Rocha et al. 2015).

### Physical analysis

#### Calorific value

Calorific value is a determining factor that allows us to know the potential of the fuel used and forms the basis of the thermal calculations of the energetic equipment in general. The gross calorific value or Higher Heating Value (HHV) is the heat produced by the complete combustion of one kilogram of fuel under specific pressure and temperature conditions, where all combustion products are reduced under the same conditions. Consequently, the water in the fuel and the resultant of the hydrogen combustion are condensed. For practical applications, it is interesting to calculate the lower heating value (LHV), which is defined as the heat produced when the water is present in the fuel, and the resultant of hydrogen combustion remains in the gaseous state. Different empirical mathematical models are available in the literature to determine these parameters. In the present research, the HHV was performed using a calorimeter bomb, the brand IKA C2000 model, according to the ASTM E711 standard norm (ASTM E711–87,1992).

#### Proximate analysis

The proximate analysis determines the fractions in weight of moisture, volatiles, ashes, and fixed carbon of one sample of material. ASTM standards for woods and residues have been used to realize the proximate analysis. The use of standard norms for determining each parameter is shown in Table 2.

**Table 2:** Techniques of analysis employed

Standard norm	Determined parameter
ASTM E 871 (2014)	Moisture
ASTM E 830 (1996) and E 1755–01 (2015)	Ash
ASTM E 872 (1982) and E 897 (2004)	Volatiles
ASTM E 872 (1982) and E 897 (2004)	Fixed Carbon

The sample was prepared according to the standard ASTM E 1757 (A. E1757–01,2011). It is valid to clarify that, in addition to performing the proximate analysis following the corresponding standard norm for the preparation of the sample of raw sugarcane bagasse, the same analysis has also been performed for each size class fraction (mean diameter) of bagasse particles obtained from the sieving analysis. The objective was to observe the influence of the particle mean diameter on the behavior of raw sugarcane bagasse's moisture, fixed carbon, volatiles, and ash contents.

The equipment used in the proximate analysis has been an analytic scale (Brand SARTORIUS Model BL210S) with a precision of 0.0001 g; a laboratory stove (Brand NOVA TÉCNICA Model NT315); a muffle (Brand EDG Model 3P-S); a desiccator; and eight crucibles.

For each of the previously calculated parameters, proximate analysis and calorific value, the standard deviation, the coefficient of variation, and the confidence intervals have been determined according to the t-test media with a 5% significance level.

#### Thermal analysis (TGA/DTG)

Thermogravimetric analysis (TGA) is a valuable tool for analyzing the transformations of chemical components when the biomass residues are subjected to a controlled increase in temperature in an inert atmosphere (pyrolysis) or oxidizing atmosphere (combustion). Experiments were carried out using the DSC-TGA analyzer TA Instruments Q600 SDT. Nitrogen was used as a purge gas with a flow rate of 20 mL/min in both experiments (inert and oxidizing atmospheres), as well as a typical sample mass of 5 ± 0.5 mg heated from 30 to 800 °C, with a heating rate of 10 °C/min.

Key characteristic parameters were calculated to analyze ignition, burnout, and combustion performance.

The ignition and burnout temperatures of biomass were identified by the intersection method presented by Morais et al., which is the most appropriate for evaluating biomasses (Morais et al. 2022). The ignition index (*D*_*i*_) represents the release performance of volatile matter in fuel and was calculated according to the equations:

3 $$D_{i} = \frac{{DTG_{max} }}{{t_{m} \, \cdot \,t_{i} }}$$

where *DTG*_*max*_ (%/min) is the maximum mass loss rate, *t*_*m*_ (min) is the time that corresponds to the maximum combustion rate, and it is the ignition time that corresponds to ignition temperature *T*_*i*_ (°C) (Vamvuka and Sfakiotakis 2011_)._

The burnout index (*D*_*b*_) was used to evaluate the burnout performance, which can be described as follows:

4 $$D_{b} = \frac{{DTG_{max} }}{{\Delta t_{1/2} \, \cdot \,t_{p} \, \cdot \,t_{b} }}$$

where $\Delta {t}_{1/2}$ is the time zone of *DTG/DTG*_*max*_ = 1/2, *t*_*p*_ is the time corresponding to *DTG*_*max*_*,* and *t*_*f*_ is the burnout time at which two consecutive points are far less than 5%.

A commonly used parameter to evaluate combustion performance is the combustion index (S); the higher this value is, the better the reactivity of the fuel will be.

The combustion index (S) is calculated using Eq. (5).

5 $$S = \frac{{DTG_{max} \, \cdot \,DTG_{mean} }}{{T_{ign}^{2} \, \cdot \,T_{burn} }}$$

DTG_mean_ is the mean mass loss rate, and T_ing_ and T_burn_ are the ignition and burnout temperatures (Moon et al. 2013).

#### Densities

The density of any biomass can be expressed in three different ways, depending on the interest or need. Sugarcane bagasse is a porous solid. Thus, it has real or skeletal, apparent, and bulk particle densities. It is important to note that sometimes, some authors modify the names of these parameters, so more attention must be paid to the definition presented in each case.

##### Real density

The real density or simply density of a biomass particle or other material is defined as the ratio between the mass of the solid particle over the solid volume of material that composes the particle, not including the pores’ volume, which eventually may be considered part of the structure of the solid particle.

6 $$\rho_{s} = \frac{{m_{p} }}{{V_{sp} }}$$

where *ρ*_*s*_ is the real density of the particle (kg/m^3^), *m*_*p*_ is the mass of the particle (kg), and *V*_*sp*_ is the volume of the particle without considering pores (m^3^).

The experimental determination of real density has been done by following the procedure reported in the standard norm ASTM D 4892-84, 1994 (*Standard Test Method for Density of Solid Pitch (Helium Pycnometer Method*). For the tests, a helium gas pycnometer has been used, brand Quantachrome ULTRAPYC model 1200e, with a precision of 10^–4^ g/cm^3^, able to make five replicate measurements on the sample, reporting the average value, as well as the standard variation and coefficient of variation.

##### Apparent particle density

The apparent particle density, considering the apparent volume, is the ratio of the particle's mass to the volume occupied by the particle and its pores. This parameter is very important when studying the fluid dynamics of bagasse particles since volume and mass will directly influence the results of simulations or any analysis. The determination of the apparent density was made using a liquid pycnometer. The method determines the apparent density from the displacement volume of a fluid caused by the addition of a solid mass. The experiment has been performed in triplicate, following the recommendations reported by Rasul et al. (1999). The apparent density can be calculated as:

7 $$\rho_{p} = \frac{1}{{V_{p} + \frac{1}{{\rho_{abs} }}}}$$

*V*_*p*_ is the volume of the pores (m^3^/kg), and *ρ*_*abs*_ is the real density of the particle (kg/m^3^).

##### Bulk density

Bulk density refers to the mass of multiple particles of a substance divided by the total volume they collectively occupy, which includes the volume of the particles themselves, the inter-particle void spaces, and the internal pore volume. This parameter holds significant importance in fuel deliveries based on volume, as it directly impacts the cost of delivering feedstock to a biorefinery and storage area. Furthermore, bulk density is not only an intrinsic characteristic of the material but is also influenced by the particle size distribution and shape. It can be described as the ratio between the mass and the volume occupied by the pores and voids formed between the particles when arranged to create a layer.

In this study, the experimental procedure used to determine this parameter has been based on standard norm ASTM E873-82 (*Standard Test Method for Bulk Density of Densified Particulate Biomass Fuels*) and in the study reported by Tannous et al. (2013) For the determination of this parameter, a laboratory electronic scale (CELTAC Brand), with precision of 0.01 g and a container with specific dimensions, has been used, where it is placed inside the biomass sample poured from a fixed height. After filling the container with biomass, its weight is measured, and the weight of the empty container is deducted from this value to determine the total weight of the biomass. To calculate the bulk density (expressed in kg/m^3^), this weight is divided by the internal volume of the container.

8 $$\rho_{bulk} = \frac{{W_{bs} - W_{b} }}{{V_{i} }}$$

*W*_*bs*_ and *W*_*b*_ are the weights of the filled and the empty containers, and V_i_ is the intern volume of the container.

These parameters have been determined for each size class fraction (mean diameters) of bagasse particles obtained from the sieve analysis, allowing one to observe the behavior of the real, apparent, and bulk densities as a function of particle diameter. In the case of raw sugarcane bagasse, the experimental values have been compared to the values determined analytically by the following equation:

9 $$\frac{{X_{t} }}{{\rho_{bag} }} = \,\sum\nolimits_{i = 1}^{8} {\frac{{X_{i} }}{{\rho_{i} }}}$$

*X*_*i*_ is the mass retained in each of the eight screens, *ρ*_*i*_ is the corresponding density of particles retained in each opening size, *X*_*t*_ is the total mass of the bagasse sample (80 g), and *ρ*_*bag*_ is the determined density.

The experimental results have been compared to the predictions of the reported correlations in the available literature. The comparison criteria adopted to validate empirical results concerning experimental values have been the Relative Mean Absolute Error (*RMAE*) defined as:

10 $$RMAE = \frac{{\left| {V_{ale} - V_{alt} } \right|}}{{V_{ale} }}\, \cdot \,100\,\left( \% \right)$$

## Results and discussion

### Geometrical analysis

#### Granulometric analysis

**Fig. 3:**
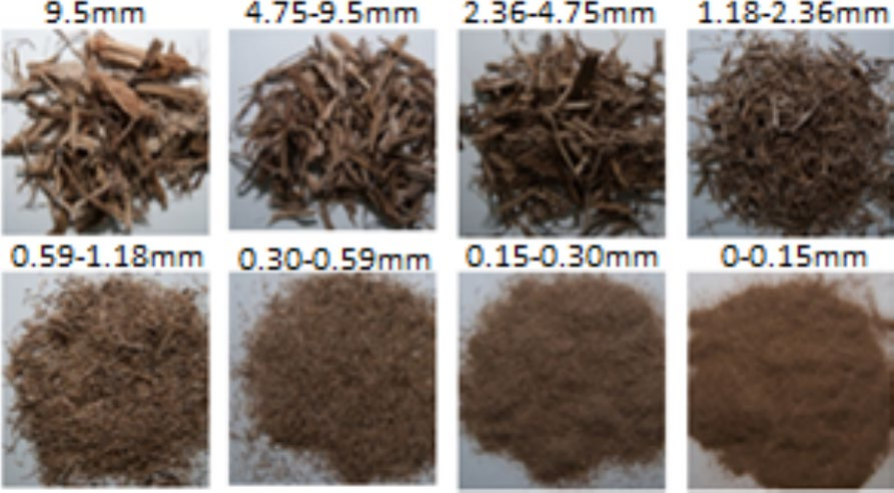
Granulometric distribution of sugarcane bagasse

**Fig. 4:**
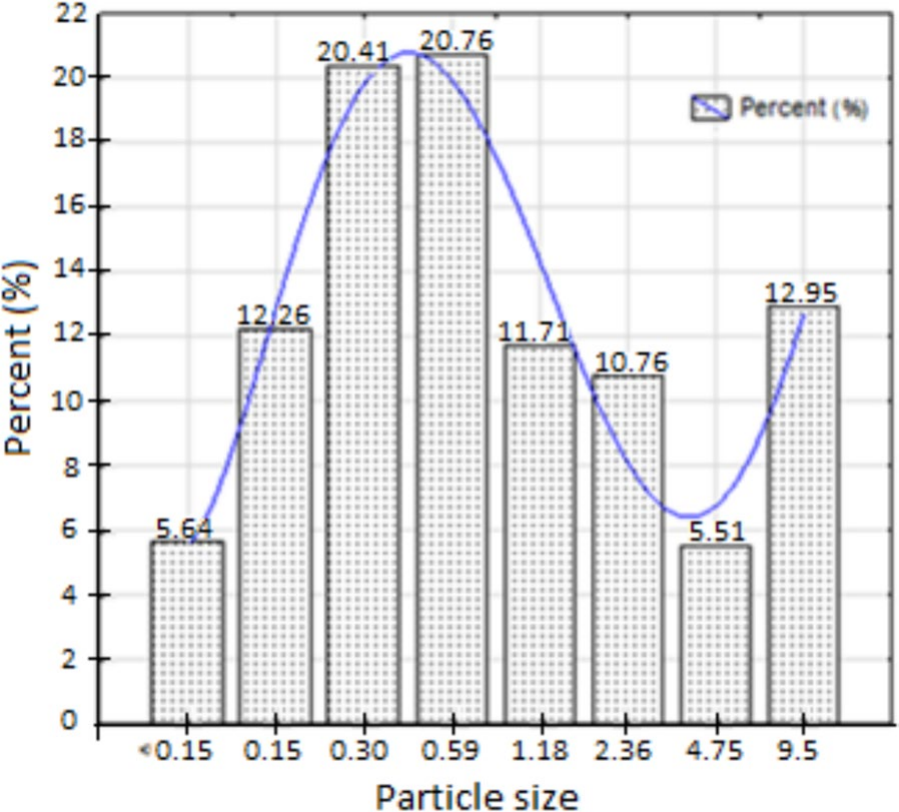
Mass percentage retained on each sieve

Figure 3 shows a picture of each fraction of the sizes of the particles obtained from the sieving analysis. For being polydisperse biomass, the characteristic dimensions have a reasonably wide distribution, including the particles in the lower end of the series of sieves; in other words, the smaller particles represented 6% of the total mass analyzed, practically the same percentage of the particles that passed through the 4.75 mm opening sieve, making the curve of Particle Size Distribution (PSD) has a concave format. The raw sugarcane bagasse PSD is shown in Fig. 4.

The characteristic diameter or characteristic dimension (*d*_*pi*_) referring to two consecutive sieves is obtained from Eq. (1), being the geometric mean diameter determined by Kunii and Levenspiel (1991), equal to 0.722 ± 0.08 mm. This diameter is referred to as the geometric mean diameter of raw sugarcane bagasse, in this case, is considered raw bagasse or bagasse “*in nature*" as the one that comes out from the productive process of the sugar mills without the application of a method of separation or pretreatment.

Figure 4 shows the behavior of PSD analysis, where the highest percentage of retained particles corresponds to sieves with openings of 0.3 mm and 0.59 mm (intermediate sieves of the set), respectively, their values being nearly four times higher when compared to the mass retained at the beginning and the end of the set of sieves. The effect of the end of the set (9.5 mm), i.e., the top of the shaker, is higher than the beginning of the sieves set (< 0.15 mm, bottom pan) because the percentage of mass retained in this sieve is higher when compared to the end of the set. Therefore, the particle mean diameter of sugarcane bagasse tends to be closer to the range of the middle of the intermediate zone towards the end of the set (0.59–9.5 mm). This behavior does not become a typical bimodal behavior as presented by other biomass particles like sugarcane trash and elephant grass particles, as analyzed in the study of Gomez et al. (2012). This behavior difference to the PSD can be due to the sample preparation method used in that study, where the biomasses were pre-processed physically by a milling process significantly different than the one used in sugar mills. However, this distribution follows a normal behavior expected for this type of analysis for sugarcane bagasse under the values reported by Gabra et al. (2001) and De Filippis et al. (2004), where the greatest concentration of particles was retained on sieves with diameters between 1.0–0.25 mm and 1.0–0.5 mm, respectively.

Various statistical parameters for particle size distribution (PSD) have been assessed for each particle size range. Parameters such as standard deviation, skewness, and kurtosis describe and classify the particulate material based on Folk and Ward's classification (Folk and Ward 1957) and the logarithmic original graphical measures classification from Blott and Pye (2001). Skewness is a measure of symmetry or, better said, of a lack of symmetry. A distribution is symmetric if it looks the same from the left and the right of the center point with skewness values near zero. In this case, the skewness of the raw sugarcane bagasse can be described as "Very coarse skewed (${S}_{kl}<-1.3$)”, using the scheme proposed by Folk and Ward (1957). The negative value of skewness represents the presence of an increased number of coarse particles (Blott and Pye 2001). This fact has been corroborated visually in the fractions of smaller sizes of particles (0–0.3 mm), where the presence of fine particles with a wide variety of distinct lengths could be observed. It is known that the standard mechanical sieving method is efficient in the "width-based separation" of particles since any particle of width is more significant than the sieve opening and cannot pass through the sieve in any orientation. However, this method presents inconsistencies in length-based separation (Igathinathane et al. 2009), allowing the finding of different particle sizes in a specific opening sieve (similar width but different length), which is very common in polydisperse biomasses that are characterized for long and fine particles with higher fiber content. The standard deviation (sorting) value measures the range, scatter, or variation in particle sizes. The determined value for raw bagasse makes it possible to classify it as "Extremely poorly sorted (${\sigma }_{l}>4.00$). This result is different from the one obtained by Igathinathane et al. (2009) for other biomasses like elephant grass and switchgrass, which have been classified as "well sorted," and from the classification obtained by El-Sayed et al. (2014), for sugarcane bagasse particles, classifying them as "poorly sorted." A possible cause of these differences may be that, in those studies, the biomasses were previously pre-conditioned through a size reduction process (fine powder) in a cutting mill machine. Kurtosis measures whether the data are heavy-tailed or light-tailed relative to a normal distribution. The values obtained for this parameter were greater than 3 (*K*_*G*_ > *3*), classifying the raw bagasse as "extremely leptokurtic." This result follows the reported by Igathinathane et al. (2009) for other types of biomasses but is different from the classification reported by El-Sayed et al. (2014), where the sugarcane bagasse particles were classified as "mesokurtic." It is important to acknowledge that these classifications may vary if the same material is studied using different sieving analysis settings, such as variations in clearance and product classification screen opening dimensions.

Knowledge of PSD is very important in thermochemical conversion processes, such as fluidized bed combustion and gasification technologies, due to its direct impact on the final gas composition and process yield. It is expected that smaller particles will have higher conversion rates, generating lower amounts of ash and tar, due primarily to the high heating rate that is achieved by the larger contact area with the gasification agent, which causes a rapid release of volatile material. In the case of larger particles, the heating rates are lower, heating slowly from the surface to the core of the particle, which may cause a temperature gradient, the core being colder than the surface, which may cause condensation or reabsorption of volatile products on the internal surface of the particle, increasing the residence time, generating greater production of ash and pyrolytic liquids, consequently decreasing the production of combustible gasses (Wang et al. 2018). Experimental studies with biomass particles have shown that when water vapor is used as the gasification agent, smaller particles produce less CO_2_ and more CO and CH_4_, and the H_2_ remains almost constant in the size range between 0.3 and 0.5 mm with a slight tendency to decrease as the particle size increases. In the case of gasification with air, the behavior is similar to the previous one (Ahmad et al. 2016).

In fluidized bed systems, achieving homogeneous and stable fluidization is fundamental for the correct operation of this equipment. Previous studies show that the mean diameter of bagasse particles larger than 3.55 mm increases the porosity of the bed due to the higher aspect ratio and low sphericity, creating preferential channels through which the gas can escape, interrupting the bubbling regime of fluidization that is one of the most used in gasification systems or fluidized bed combustion (Yang 2003). It is expected that a decrease in porosity as the diameter of bagasse particles decreases to about 1.0 mm, which could represent an increase in the combustion velocity in the bed when porosities between 0.5 and 0.6 are achieved (Bin Yang et al. 2005). In addition, larger particles tend to crosslink with high cohesive forces, creating larger spaces through which the inert material of the bed and the air can easily pass, reducing the contact area and the particle–particle collisions, affecting the excellent mixing and homogenization of the bed as well as the final gas composition (Chok et al. 2010). Higher gas surface velocities should be used to break these solid inter-particle bonds resulting from Van der Waals forces to avoid this phenomenon. Studies of cold fluidization of bagasse particles (Pérez et al. 2017) show that particles with average diameters between 3.55 and 1.77 mm present ratios of complete and minimal fluidization velocities (V_cf_/V_mf_) in the order of 1.43–1.55, ensuring a complete mixing of the bed with less segregation. The V_mf_ tends to decrease at high temperatures, and the V_cf_/V_mf_ ratio remains practically constant above 800 K.

According to the criteria previously exposed, we can conclude that sugarcane bagasse particles with average diameters between 3.55 and 1.0 mm should ensure a good mixture and homogenization of the bed, obtaining high conversion rates and consequently better yields in the thermochemical conversion processes. It is worth clarifying that in the specific case of sugarcane bagasse particles, a decrease in particle size can cause an increase in ash content due to the presence of inorganic soil contaminants and oxides from the milling process in sugar and alcohol mills, as can be seen in "Proximate analysis" section.

#### Geometric analysis

The results of the geometric analysis are presented in the first study developed by the authors due to the extension and importance in the design and performance of thermochemical conversion systems (Pérez et al. 2019).

The study's conclusion revealed that sugarcane bagasse's particle size distribution resembles other biomasses with similar physical traits. The dissimilarities from other biomass types mainly arise from variations in the disintegration mechanisms of particles, which directly depend on the material's internal structure. Smaller-sized bagasse particles demonstrated a lower aspect ratio (length/width) with average dimensions of length (4.705 ± 3.511 mm) and width (1.101 ± 1.235 mm) for raw bagasse. The aspect ratio varied between 2.14 and 5.5 for different particle sizes, with an average value of 3.922 ± 2.736. Larger-sized particles showed significantly different aspect ratio values across different biomass types, but as the diameter decreased, these values tended to converge around 2.0–2.5, regardless of the biomass kind. This convergence is primarily due to the differences in particle size reduction. The average sphericity value ranged from 0.272 to 0.558 across the granulometric ranges analyzed, with a mean value of 0.3975 for raw bagasse. The study also developed approximate mathematical models using non-linear regression to determine raw bagasse particles' characteristic aspect ratio and sphericity. These correlations are applicable within a specific range of class sizes of the sieves, i.e., smaller than 9.5 mm and greater than 0.075 mm.

### Chemical analysis

#### Ultimate analysis

The results obtained from the ultimate analysis are shown in Table 3, and other results are reported by different authors working with the same biomass type.

**Table 3:** Results of ultimate analyses of dry and ash-free sugarcane bagasse

References	Rocha et al. (2015)	Virmond et al. (2012)	Bizzo et al. (2014)	Galina et al. (2018)	Rocha^*^ et al. (2015)	This work^**^
Ultimate analysis wt.% (daf)						
Carbon (C)	50.57	45.0	42.61	49.2	44.9	45.7
Hydrogen (H)	6.05	6.05	5.09	5.76	6.1	6.37
Nitrogen (N)	0.73	0.3	0.63	0.48	0.27	0.28
Oxygen (O)	42.55	48.57	50.9	44.56	48.74	47.56
Sulfur (S)	0.1	< 0.01	0.12	–	–	0.09
Chlorine (Cl)	–	0.05	0.1	–	–	–
Phosphorus (P)	–	0.03	–	–	–	–

^*^In Rocha et al. (2015) sixty sugarcane bagasse samples were collected and analyzed from São Paulo state, and the average results were presented

^**^Sugarcane bagasse “*in nature*” prepared according to the ASTM D3176-3179

Knowing the H/C and O/C ratios for thermochemical conversion processes is more important than the separated knowledge of the biomass's H, O, and C elements. The H/C and O/C molar ratios in biomass are generally higher than those found for coal, ranging between 1.3–1.5 and 0.5–0.6, drastically reducing the calorific value of these fuels (Virmond et al. 2012). The energy a fuel can supply is due to the positive balance between the energy needed to break the bonds and the energy released to form new bonds. Considering this and knowing the high enthalpy values of the bonds H-C and O-C, greater energy is needed to break them, translating into less energy available. The C–C bonds have a lower enthalpy, which contributes to a better energy balance, and these C–C bonds are the majority in coals, which increases their HHV compared to biomasses (Braz 2014). The values obtained in this work for H/C and O/C ratios were 1.4 and 0.67, respectively.

The contents of Carbon (C) and Hydrogen (H) correspond to the fuel fractions that contribute to the increase in the HHV. Oxygen (O), unluckily, does not contribute to heating value and makes it challenging to transform biomass into liquid fuels, as in the Fischer–Tropsch synthesis case. Another problem that high oxygen content can bring is consuming part of the hydrogen contained in the biomass, producing water. Thus, high H/C content does not necessarily translate into high gas yield (Basu 2013). However, oxygen acts positively by promoting homogeneous oxygenation, which improves the conversion rate and efficiency of the solid fuel transformation into syngas (Virmond et al. 2012). The presence of N in the biomass composition brings about the formation of nitrogen oxides during the thermochemical conversion processes. This element can react with oxygen in the air, forming NOx (90% NO, 10% NO_2_, and N_2_O in small quantities). The NOx formation from the oxidation of fuel N is the most important mechanism to be considered in practical applications of gasification or combustion units due to emissions that can generally exceed the established limit, contributing to the greenhouse effect and the occurrence of acid rain (Rocha et al. 2015). In addition, the N content has a negative impact on HHV. Concentration values of N on fuels above 0.6 wt% on a dry basis can result in emission problems (exceeding the regulation of emission limits) in thermochemical conversion processes (Virmond et al. 2012). The sulfur (S) and chlorine (Cl) contents of lignocellulosic biomass are exceptionally low, which is a significant advantage in its utilization in energy conversion systems when SO_2_ emission problems are taken into account (Basu 2013). The importance of S and Cl in the composition of materials is not primarily related to atmospheric emissions but to industrial plants' corrosion processes (Sommersacher et al. 2012). During gasification or combustion processes, sulfur, initially present in biomass, is transformed into gas. The release of high concentrations of SO_2_ gas, besides being converted to H_2_S, in contact with the alkali metal oxides (CaO and MgO) causes sulfation, that is, the retention of this gas in the form of sulfate, and this process leads to the release of chlorine (absorbed by biomass through fertilization) causing corrosion of the system by the formation of iron chloride and zinc chloride formed on metal surfaces, which are highly corrosive compounds, causing serious damage to heat exchangers systems used for cooling the syngas, and on the operation of gas turbine blades in the gasification-based power generation cycles (Virmond et al. 2012).

Furthermore, it must be considered that these two compounds significantly impact the cost of sulfur removal in recovery units. Thus, it has been reported that there are two significant properties to consider when designing thermochemical conversion systems. Fuels with concentrations of S above 0.2 wt.% dry basis can emit acid gasses, such as hydrogen chloride (HCl) if chlorine-derived compounds are present in the biofuel composition and others if lead or other metals are present (Virmond et al. 2012).

#### Compositional analysis

Lignocellulosic biomass is a complex mixture of natural polymers of carbohydrates known as cellulose and hemicellulose, in addition to lignin and small amounts of other substances, such as extractives and ashes, which are contained in the cell wall of plants (Ungureanu et al. 2022). The compositional analysis allows us to determine the percentage of these elements in the biomass. There are three categories of compositional analysis methods: sulfuric acid hydrolysis, near-infrared spectroscopy (NIRS), and kinetic analysis. The most used method is based on a two-step sulfuric acid hydrolysis, which has been used with some specific modifications introduced for researchers for different objects and conditions for over a century (Cai et al. 2017).

Table 4 shows the results obtained by applying two different techniques to implement the sulfuric acid hydrolysis method. The compositional analysis revealed that the percentage of the main components does not differ significantly between the samples analyzed. The content of lignin ranged in a very narrow interval (21.1–22.85%), while the ranges for hemicelluloses (24.01–28.9%) and cellulose (35.31–45.5%) were relatively wider. However, the content of extractives displayed a higher dispersion. A possible reason may be the extraction method reported by Bizzo et al. (2014) and Rocha et al. (2015), where some of these extractives may be sucrose. The inconsistencies between the results reported by the authors can be derived from the different analysis techniques used.

**Table 4:** Analytical results of compositional analysis of sugarcane bagasse

References	Means components			
	Cellulose	Hemi-cellulose	Lignin	Extractives
Rocha^*^ et al. (2015)	42.19	27.60	21.56	5.63
Rocha^**^ et al. (2011)	45.50	27.0	21.10	4.60
Rocha et al. (2015)	41.8	28.9	21.4	15.0
Bizzo et al. (2014)	35.31	24.01	22.85	14.7

^*^sixty samples of pre-treatment sugarcane bagasse from the São Paulo sugar mill. The average of the results are presented

^**^raw sugarcane bagasse

On the other hand, in general, the results of the composition of the main components (cellulose-hemicellulose-lignin) are in good agreement with previous results reported in the literature, within the ranges of 40–50% for cellulose, 25–30% for hemicelluloses, and 20–25% of lignin (Ungureanu et al. 2022; Antonio Bizzo et al. 2014). Another important conclusion is that regardless of the variety and type of sugarcane used, the chemical composition of the bagasse is very similar, with insignificant variations between one sample and another, as has been demonstrated in Rocha et al. (2015).

Regarding sugarcane bagasse composition, it may represent an advantage in applying this material as a solid biofuel for thermochemical conversion processes since cellulose contributes to the fixed carbon content of the material and presents more excellent thermal stability than hemicellulose. Nonetheless, the thermochemical decomposition of cellulose is a major contributor to tar production in the pyrolysis or gasification of biomass (Toscano Miranda et al. 2021). The percentage of lignin present in the material is directly related to its heating value, given that lignin is a major contributor to the energy potential presented by solid biofuels in thermochemical conversion processes, the higher its value, the better, in addition to providing a slower burning rate of fuel, due to its high thermal resistance (Raj et al. 2015). The extracts present in the lignocellulosic material collaborate positively with the heating value of the solid biofuel since they have energetic content in their constituent molecules released during combustion. Although cellulose and hemicellulose are in a higher percentage (around 70%), the heat energy generated during the burning of bagasse is low compared to lignin. It can be concluded that the lignin present in the fuel is present in a small amount of the total dry matter. However, it is responsible for the greater energy potential generated in the boilers of the power plants. The content of hemicellulose and cellulose provides an estimate of the degree of thermal degradation of the biomass. Higher content means they are easier to degrade at low temperatures (Toscano Miranda et al. 2021).

### Physical analysis

#### Calorífic value

In this case, the HHV has been determined only for sugarcane bagasse in its natural form (raw bagasse) with equilibrium moisture content (8.71%), and the result determined was 16.08 ± 0.32 MJ/kg. This value practically coincides with the value found by Rocha et al. (2015), in which they analyzed more than 60 sugarcane bagasse samples from different regions of Brazil, reporting a mean value of 16.01 MJ/kg. However, compared to other studies, this value is slightly lower, which may be determined by the variety of sugarcane used, soil type, climate, and other factors.

Using Eq. (11) developed by Makray (1984), which relates the HHV on a dry basis with the LHV on a wet basis, it is possible to obtain the LHV, knowing the fraction by weight of hydrogen in the biomass on a dry basis (6.1%) (Rocha et al. 2015) and its moisture on a wet basis. In this equation, the moisture of the biomass, the energy required to vaporize this moisture, and the latent heat of the vaporization process of the water formed during the combustion are discounted from the HHV.

11 $$LHV = \left( {HHV} \right)\, \cdot \,1 - \frac{W}{100} - 22,1\, \cdot \,H_{db} - 0,442\, \cdot \,\left[ {\left( \frac{W}{18} \right) - \left( {\frac{{H_{db} \, \cdot \,W}}{2}} \right)} \right]$$

This parameter is the essential property of a fuel, which determines the energy value that it can have. The energy density of a certain species can be evaluated by combining the amount of biomass produced per unit area with its high heating value. In the case of the sugarcane bagasse, this value can be expected in the range of 229.2 GJ/ha.yr (Rocha et al. 2015). The HHV measured is compatible with values found in the literature for lignocellulosic biomass and finds itself in the order of expected magnitude (Toscano Miranda et al. 2021).

#### Thermal analysis (TGA/DTG)

The TGA and DTG characterization is helpful for several reasons, including understanding the grade of reactivity of a given biomass and its tendency to form carbon and volatile matter. In addition, it causes the biomass's weight loss with the increase in temperature, which serves as an indicator for optimizing the gasification and pyrolysis processes. Some researchers also use these analyses to determine biomass's moisture, cellulose, hemicellulose, and lignin contents (Cai et al. 2017). The decomposition of lignocellulosic biomass is a complex process composed of a series of consecutive chemical reactions that compete, characterized by four fundamental stages.

**Fig. 5:**
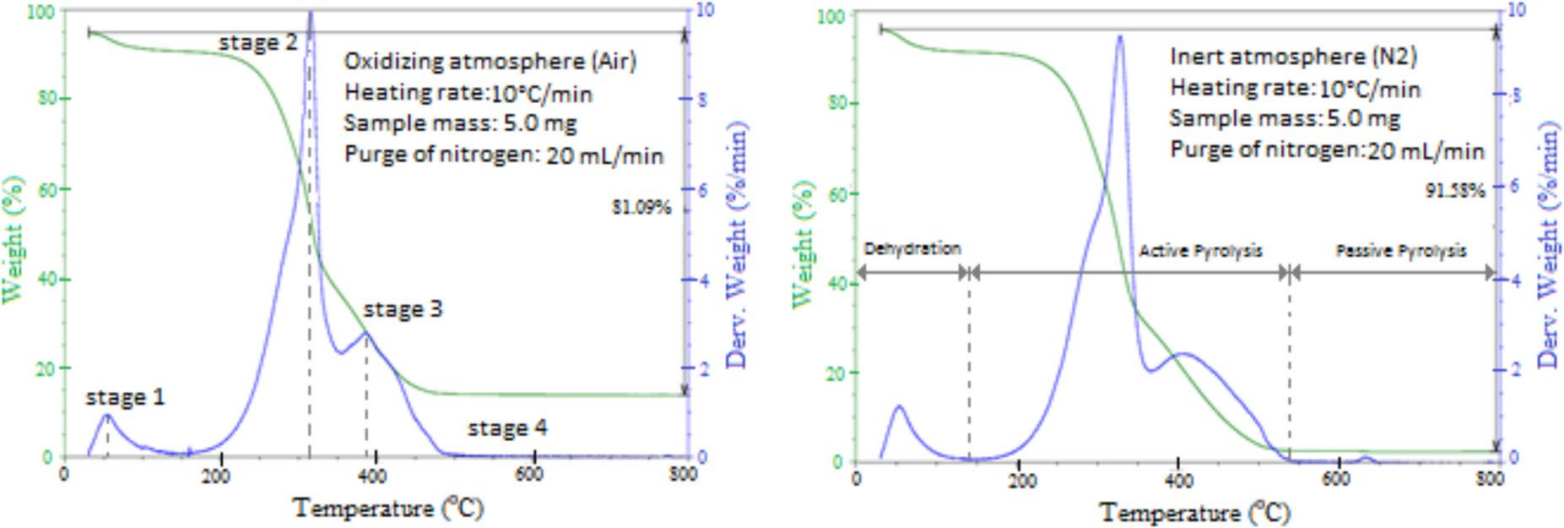
Differential thermogravimetric analysis

Figure 5 shows the thermogravimetric (TGA) and derived thermogravimetric (DTG) curves of the biomass samples obtained in an air and nitrogen atmosphere. The first event (stage 1) occurs at a temperature below 100 °C. It is related to the moisture loss in the material, corresponding to the drying phase between 50 and 150 °C, with an approximate weight loss of 5–8%. The maximum degradation temperature in this stage was observed from 52 to 55 °C. The second event (stage 2) is related to the decomposition of the organic matter in the biomass, and the degradation occurs between the temperatures of 150 and 360 °C. It can be attributed mainly to the decomposition of hemicellulose and cellulose compounds. Lignocellulosic components decompose at different rates; hemicellulose has a random, amorphous structure with short side chains, little strength, and a lower degree of polymerization, which makes it less thermally stable than cellulose, which is the main component of the biomass cell wall, associated with long-chain semi-crystalline structure with a high degree of polymerization (Raj et al. 2015). From Fig. 5, a main peak with an imperceptible shoulder is observed between 160 and 300 °C, which is typically considered to cause hemicellulose decomposition due to its less stable structure, while the main peak between 300 and 360 °C is considered to be mainly due to the degradation of cellulose, characterizing the process of devolatilization and degradation of volatile matter, that appears to occur simultaneously. The gases released in this process mix easily with oxygen and burn more easily than fixed carbon. This is a fast-burning region, where the mass loss rate reaches its highest value from 47 to 54% in both environments. The maximum degradation temperature of cellulose is observed from 310 to 330 °C. The third stage involves the degradation of the residual volatiles and fixed carbon, which occurs from approximately 360–540 °C, with a mass loss of 29–31%, respectively. At 390–410 °C, there is a peak temperature related to the maximum decomposition rate of residual lignin, which starts at approximately 200 °C and ends at 600 °C according to previous studies reported in the literature (Morais et al. 2022), this more extensive temperature range of lignin decomposition is because of the existence of bonds between lignin and cellulose and hemicellulose. The cracking of lignin is much more difficult than breaking the glycosidic bonds in cellulose and hemicellulose (Najafi et al. 2023). Lignin is an integral part of the secondary cell wall of plants, being a complex, highly branched polymer of phenylpropane, is heavily cross-linked and has very high molecular weight, hence has a high thermal stability as compared to cellulose and hemicellulose (Raj et al. 2015). The fourth stage corresponds to the complete decomposition of the material, which occurred at 510 and 540 °C, respectively.

Two different pyrolysis regions were observed during thermal degradation in an inert environment. After the stage of loss of moisture, a sharp drop in weight loss is observed; this region corresponds to the active pyrolysis zone. The second pyrolysis zone is characterized by insignificant changes in the weight loss curve, called the passive stage. The analysis of the weight loss rate curve shows in the pyrolysis active zone two different peaks that can be associated with the degradation of hemicellulose and cellulose in only a merged peak and a residual lignin peak. The first weight loss appears at a temperature of 140 °C due to the evaporation of water. The temperature for initiation of active pyrolysis occurs at approximately 150 °C and ends at 540 °C. Table 5 shows the most relevant parameters from combustion and pyrolysis experiments, concluding that approximately 81 and 91.5% of the material was oxidized in both environments.

**Table 5:** Relevant parameters from combustion and pyrolysis experiments

Atmosphere	Stage	T_i_ °C	T_onset_ °C	T_endset_ °C	T_f_ °C	T_peak_ °C	wt loss %	T_ign_ °C	T_burn_ °C
Air	1	35	43	102	148	52	5.0	275	450
	2	160	255	340	353	320	47.0		
	3	360	370	480	510	390	29.0		
	4	510	–	–	800	–	0.1		
Nitrogen	1	35	44	100	148	55	6.3	285	495
	2	150	260	350	365	340	54.1		
	3	375	390	510	540	410	31.0		
	4	540	–	–	800	–	0.1		

Figure 5 and Table 5 show that the TG and DTG curves and different characteristic temperatures of each process exhibited similar behaviors but with their characteristics in each atmosphere. Combustion and pyrolysis profiles display different patterns of devolatilization. The pyrolysis experiments showed the most considerable temperature decomposition interval of lignocellulosic material, starting at 150 °C and ending at 540 °C. Nevertheless, the combustion profile has a main peak that is higher and more symmetrical than in the pyrolysis profile but occurs at a lower temperature. These differences in the devolatilization profiles in an inert or oxidizing atmosphere can be attributed to the presence of oxygen in cross-linking reactions at the surface of the particles, which would cause a fast devolatilization. When reacting oxygen reaches the surface of the particles before they begin to decompose thermally, a significant part of the volatile matter is consumed at low temperatures through heterogeneous combustion or oxidation reactions that will transform the volatiles into other easier compounds to eliminate at lower temperatures. Some authors describe this region as burning in which volatiles are released and burned quickly (Munir et al. 2009). Hence, it can be affirmed that the thermal decomposition process under an oxidative atmosphere occurs earlier than at pyrolysis conditions. Another possible explanation for the difference in profiles is that at higher temperatures where secondary combustion occurs, the reaction tends to be controlled by intra-particle diffusion (Silva Filho and Milioli 2008). Thus, oxidizing atmospheres using moderate heating rates tend to restrict the diffusion of oxygen to penetrate the molecular network of char. It causes varying oxidation rates of carbonaceous matter from the outer to the center of particles, mainly due to forming an outer edge of highly cross-linked oxidized material characterized by reduced porosity. This fact produces an accumulation of oxygen in the mesopores. As a result, the burning time increases, and burnout is reduced. It can be concluded that oxygen has more difficulty accessing micropores and tortuous channels within the particle in oxidative atmospheres than in the inert N_2_ atmosphere; this increases combustion in these areas. In addition, this fact explains that weight loss is higher than registered in an oxidative medium and, consequently, less residual ash is generated (López et al. 2014a). In both environments, the same behavior has been observed at low temperatures, where the dominant processes are the release of moisture and the decomposition of hemicellulose compounds. This indicates that the desorption of volatiles is independent of the reacting atmosphere used (Morais et al. 2022).

Ignition and burnout temperatures are two crucial properties of fuels that play an essential role in the selection, safety storage, and reaction degree. Therefore, these two parameters must be carefully analyzed when selecting any technology or thermochemical conversion equipment. The ignition temperature of bagasse obtained in the thermal analysis was between 275 and 285 °C, and the burnout temperature was between 450 and 495 °C in both environments, respectively, being higher in the inert atmosphere due to the less reactivity of the biomass. These two parameters are in the expected range, considering the operating conditions defined above and by previous studies (Morais et al. 2022). Table 6 shows the main characteristic parameters of fuel combustion, which are very important in selecting thermochemical applications.

**Table 6:** Characteristic parameters of combustion of different fuels

References	Atmosphere/fuel	*D*_*i*_* (%/min*^*3*^*)*	*D*_*b*_* (%/min*^*4*^*)*	*S (%*^*2*^*/min*^*2*^°C^3^*)*
Galina et al. (2018)	O_2_/N_2_ bagasse	–	–	14.5∙10^–8^
	Coal	–	–	1.83∙10^–8^
Lopez et al. (2014b)	O_2_/CO_2_ Sunflower	9.66∙10^–3^	86.0∙10^–4^	–
	Rape	7.45∙10^–3^	38.8∙10^–4^	–
	Corn	1.78∙10^–3^	4.37∙10^–4^	–
Protásio et al. (2017)	O_2_/N_2_ Babassu nut shell	25.7∙10^–3^	–	17.8∙10^–7^
Wang et al. (2011)	Coal	–	–	2.23∙10^–8^
Qiang et al. (2012)	O_2_/N_2_ Semicoke from coal	10.10∙10^–3^	9.78∙10^–4^	12.5∙10^–8^
This work	O_2_/N_2_ Bagasse	13.52∙10^–3^	104.5∙10^–4^	11.1∙10^–8^
	N_2_ Bagasse	11.59∙10^–3^	86.68∙10^–4^	6.71∙10^–8^

The volatile matter (83.98%) and fixed carbon (12.56%) of bagasse are under proximate analysis because volatiles will ease the quick ignition and effective combustion; thus, the combustion index (S) is directly proportional. Besides, the compositional analysis further indicates that with its lignin (21–23%), bagasse introduces thermal stability, while cellulose and hemicellulose introduce reactivity. The high heating value of 16 MJ/kg indicates that the obtained S index is some measure of the energy capacity of the material. Results of the obtained CHNS/O analysis indicate cleaner and more efficient combustion due to the low nitrogen and sulfur levels than other biomass fuels such as oil palm trunks and fronds, empty fruit bunches, palm heart shells, rice husks, rice straw, and kenaf biomass, which have a higher N and S content in their composition (Sohni et al. 2018).

The reactivity of biomass depends mainly on the content of volatile matter and fixed carbon in its composition (López et al. 2014a). As the fixed carbon fraction, which burns in the solid state, increases in the fuel, it provides more excellent thermal stability and less biomass weight loss during combustion. A high content of volatile matter means a high content of hemicellulose + cellulose, which promotes greater devolatilization, increasing the rate of thermal decomposition at lower temperatures due to the low thermal resistance of these compounds, causing greater reactivity in fuel biomass when compared to carbon (Virmond et al. 2012). A high H/C ratio and a high O_2_ content in the chemical composition of the fuel improve thermal devolatilization and oxidation at a high rate, therefore improving the ignition characteristics (Liu et al. 2013). Considering the above, biofuels with a lower ignition temperature is easier to ignite, and biofuels with a lower burning temperature value have better-burning efficiency. These characteristics favor a higher ignition index (*D*_*i*_) and a higher burnout index (*D*_*b*_). The characteristic combustion index (*S*) reflects the reactivity of the carbon throughout the oxidation reaction, and the higher this value, the better the combustion performance. Therefore, a higher combustion index (*S*) means better performance in the ignition and burning processes, which will occur at lower temperatures and in shorter times (Liu et al. 2013).

Table 6 shows that the bagasse analyzed in this study has ignition index (*D*_*i*_) and Burnout index (*D*_*b*_) values higher than fossil fuels such as coal and that most of the other biofuels reported in previous studies. This fact can be justified due to the more significant and faster emission of volatile material, factors that effectively contribute to the acceleration of fuel ignition at lower temperatures. Therefore, an increment in the Volatile Matter/Fixed Carbon ratio, with the decrease in the final carbonization temperature, explains the decrease in time and the ignition rate, corresponding to shorter combustion times. An increase in burnout temperature causes a decrease in reactivity. Consequently, biochar produced at lower temperatures will be easier to ignite and perform better (Qian et al. 2012). However, the ignition index of babassu nutshell is much higher than bagasse's; this occurs because its ignition (240.35 °C) and burning (433.86 °C) temperatures are lower than those reported for bagasse. Therefore, a shorter ignition time is expected (22.8 min) (Protásio et al. 2017) when compared to the bagasse ignition time (25 min), which favors an increase in the ignition index. The combustion index (*S*) obtained is very similar as reported by Qian et al. (2012) for semicoke obtained from coal at a pyrolysis temperature of 500 °C, and very close to the value reported by Galina et al. (2018) for sugarcane bagasse obtained from the same region as the sample used in this study. As the pyrolysis temperature for biochar production increases, the ignition temperature and the combustion temperature tend to increase, which also causes the combustion index to decrease. In other words, biochar pyrolyzed at lower temperatures will have a better ignition performance and a better combustion reactivity, burning more easily and strongly (Qian et al. 2012). However, great care must be taken when comparing combustion characteristic parameters since they strongly depend on the physical and chemical properties of each type of fuel and the equipment and operating conditions in which the thermal analyses are carried out. It is known that an increase in the heating rate generally causes an increase in the values DTG curve of the main peak of weight loss, which will influence the ignition and burnout temperature values and, consequently, the characteristic combustion index and ignition and burnout index (Morais et al. 2022).

#### Proximate analysis

The average values determined for each of the ranges of particle size obtained from the sieve analysis are presented in Table 7.

**Table 7:** Main results of proximate analysis

Sieve	wt % wet basis	wt % dry basis		
size (mm)	Moisture (%)	Ash (%)	Volatile (%)	Fixed carbon (%)
9.5	8.63 ± 0.27	1.36 ± 0.09	86.10 ± 1.53	12.54 ± 1.60
4.75	7.24 ± 0.11	1.69 ± 0.10	84.00 ± 0.40	14.31 ± 0.37
2.36	8.40 ± 0.23	1.86 ± 0.07	87.90 ± 1.39	10.24 ± 1.45
1.18	8.40 ± 0.23	2.56 ± 0.31	84.20 ± 2.48	13.24 ± 2.22
0.722^*^	8.71 ± 0.40	3.56 ± 0.82	83.98 ± 1.48	12.56 ± 1.11
0.59	7.85 ± 0.28	2.51 ± 0.25	84.60 ± 1.91	12.89 ± 1.68
0.30	7.76 ± 0.08	5.32 ± 0.39	83.80 ± 1.04	10.88 ± 1.38
0.15	6.39 ± 0.24	11.39 ± 0.82	83.07 ± 1.46	5.54 ± 1.96
< 0.15	6.76 ± 0.23	18.20 ± 0.35	76.67 ± 1.73	5.13 ± 2.29

^*^bagasse "in nature" (composed of all sieve fractions and with geometric mean diameter ${\overline{\text{d}}}_{\text{p}}$)

Table 7 shows that the equilibrium moisture slightly tends to decrease as far as it reduces the mean particle diameter, ranging from 8.63 to 6.76%. For the bagasse "*in nature*," the moisture content was 8.71 ± 0.4% with a standard deviation of 4.65%. These obtained values agree with the values reported by Virmond et al. (2012), corresponding to equilibrium moisture content at the pressure and ambient temperature.

The high moisture content typically found in sugarcane bagasse (≥ 50%) (Toscano Miranda et al. 2021) has, as its main negative effect, the decrease in the heating value of the fuel since a certain amount of energy is used to evaporate that moisture. Also, it can cause ignition problems in combustion and gasification processes and influence the composition and quality of the pyrolysis products, which is the first step of these processes. Another problem that the high moisture content can cause is that it shortens the space and the sustainability of the flaming, which causes the diminution of radiation in tube walls of the heat exchangers and boilers, increasing the time and energy required and the amount of unburned materials, producing a higher rate of particulate deposition on the surfaces (Al-Shemmeri et al. 2015). In typical sugar mills where the bagasse is stored in piles, stirred, and regularly exposed to the sun, the moisture can be reduced to 10–25% in equilibrium with the relative air humidity (Toscano Miranda et al. 2021). The behavior of this parameter as a function of particle size shows an expected result since a decrease in size favors compaction, reducing interparticle spaces and, therefore, the possibility of absorption of ambient humidity. This should be considered for assessment of the cost of transportation or energy penalty in drying the biomass in thermochemical conversion applications. Most studies determine an average cost for harvesting and transporting biomass between 10 and 40 US$/ton, depending on the range assumed, generally between 50 and 130 km, confirming that a higher moisture content negatively affects the biomass supply chains (Woo et al. 2018).

The volatile matter content allows us to get an idea of the facility of ignition and the burning rate of the solids that, along with the fixed carbon/volatile material ratio, determines the stability of the flame during the combustion. It is known that biomasses have a high reactivity due to their physical and chemical characteristics. Volatile matter is composed of lignocellulosic material composed of three constituents: cellulose, hemicellulose, and lignin, representing approximately 85% of the total mass of volatile matter (Al-Shemmeri et al. 2015). The sugarcane bagasse has a high cellulose content, approximately between 40 and 50%, so it is expected to have a high rate of combustion compared to other fuels such as coal; however, the energy content is less due to the low lignin content, between 20 and 25%. In this case, the content of volatile matter and fixed carbon for the sugarcane bagasse "*in natura*” was 83.98 ± 1.48% and 12.56 ± 1.11%, respectively. These values do not present much variation when compared to the other ranges of sizes of particles analyzed, being very similar to small variations in the range of sizes of 0.3–9.5 mm, concluding that the size of particles does not influence the final mass of volatile matter generated. Only in the small particles with diameters ≥ 0.15 mm were the variation of volatile matter and fixed carbon greater, with a tendency to decrease, which is logical to expect due to the smaller mass of these particles. As for the influence of these parameters in the ignition front speed and the burning rate, it can be assumed, based on the results reported by Ryu et al. (2006), that larger particles will have an ignition front speed higher due to the decrease of its bulk density. In contrast, the burning rate tends to increase linearly with the decrease of this parameter. The explanation of this hypothesis may be that larger particles will have a greater void between them, forming channels through which air or gas flow can easily pass through them, causing a greater ignition front speed around these channels. It also causes large fluctuations in the temperature profile and chaotic burning patterns. This would not be an expected common behavior where larger particles with higher bulk density tend to have slow burning rates and lower ignition front speeds (Ryu et al. 2006). However, more detailed studies must be carried out to verify this fact. A negative factor of fuels with high levels of volatiles is the presence of an accelerated burning rate, since the eliminated gasses, in the form of a flame, propagate the heat through a large burning area, preventing high temperatures in the furnaces from being reached by the material. However, a high content of volatile material that is volatilized and later condensed is important in pyrolysis processes since a higher content of volatile matter implies a higher yield of produced gas or bio-oil (Raj et al. 2015). In general, the results obtained in the Proximate Analysis, the contents of moisture, ash, volatile, and fixed carbon were in agreement with the ranges previously described in the literature (Sudagar et al. 2020), specifically for the case of considering only Brazilian sugarcane bagasse, the results were very close to those reported by Bizzo et al. (2014), Virmond et al. (2012) and Rocha et al. (2015).

On the other hand, the ash content increases as the mean diameter of particles decreases, leading to a considerable increase of ash content for particles with a size equal or lower than 0.15 mm, which can be explained by the existence of higher quantity of inorganic contaminants from the soil that has small granulometric size, such as mechanical contamination with iron and titanium oxides coming from the rolls, due to the consequence of the process of juice extraction and milling of sugarcane. Regarding the ash content for raw sugarcane bagasse, the value was 3.56 ± 0.82%. This value is slightly higher than those reported by Bizzo et al. (2014). This difference may be influenced by the sugarcane varieties used, soil type, time and method of harvest, weather conditions, and other factors. However, it is an acceptable value for this type of biomass compared to other works reported in the same research, which is practically equal to the one reported by Rocha et al. (2012) in a research carried out in the same region where this work was developed.

Ash content is a significant factor to consider in energy applications since a high percentage of this parameter leads to fouling and deposits on the heat transfer surfaces of equipment, slagging, and corrosion problems. Two parameters are essential in analyzing the ash: its chemical composition and fusibility characteristics. Correlations reported in the literature allow the evaluation of the tendency toward the formation of deposits of molten ashes in processes of co-combustion of coal and biomass. A relationship frequently used considers the ratio of the concentration of basic oxide components to the concentration of acidic oxide components B/A_ratio_ in the composition of the residue, considering the presence of phosphorus in the form of P_2_O_5_. The value of this ratio indicates the tendency of deposits’ formation: for B/A_ratio_ < 0.75, the tendency of ash deposit forming is lower, and for B/A_ratio_ > 0.75, the tendency is higher, indicating that the greater the concentration of basic components, the greater the risk of fused ash deposits (Fioranelli and Bizzo 2023; Teixeira et al. 2012). Nevertheless, this index must be used very carefully in biomasses that contain potassium, which is one of the leading causes of slagging and fouling problems, so its determination without considering this element could be unrealistic (Fioranelli and Bizzo 2023). Other correlations consider the content of Cl and S when they are present in the biomass composition, which has a strong fouling tendency to the surfaces, causing corrosion. The application of indexes based on sulfur content (R_s_) showed low slagging potential when R_S_ < 0.6; when 0.6 < R_S_ < 2, it is medium; for 2 < R_S_ < 2.6, it is considered high and extremely high for R_S_ > 2.6. However, this index may also produce incoherent predictions because the biomass has a much lower sulfur content than coal. If chlorine content in the fuel were Cl < 0.2, representing a low slagging inclination, 0.2 < Cl < 0.3 medium, and Cl higher than 0.3, the tendency is higher (Pronobis 2005). One indicator used in recent years to predict fouling and slagging is the alkali index (*AI*). The alkali index expresses the quantity of alkali oxides, such as K_2_O and Na_2_O, in the fuel per unit of fuel energy (kg alkali/GJ). For herbaceous fuels, like miscanthus, sugarcane bagasse, and other similar biomasses, problems of agglomeration, slagging, and fouling are expected to occur when the value of the alkali index is near or higher than 0.34 kg alkali/GJ (Fioranelli and Bizzo 2023). For biomasses, using the fouling index (Fu), which is based on the Base-to-Acid ratio, may be adequate because it gives more relevance to the alkaline elements, which are the main fouling agents. When F_u_ < 0.6, the fouling potential is low, medium for 0.6 < F_u_ < 1.6, high for 1.6 < F_u_ < 40, and extremely high with a tendency to deposit sintering when F_u_ > 40 (Fioranelli and Bizzo 2023). Table 8 shows the main components obtained from the ash analysis of Brazilian sugarcane bagasse. The first two columns correspond to the results of ash obtained in the laboratory in an electric furnace. Column 3 shows the results of industrial fly ash, the finest powdery particles that “fly up” from the biomass combustion chamber and are captured in the gas outlet region through emission and particulate control systems. The last two columns show the composition of bottom ash, which is too large to carry with the flue gases and deposit on the walls or fall to the bottom of the furnace.

**Table 8:** Results of ultimate analyses of sugarcane bagasse ash

References	Bizzo^A^ et al. (2014)	Virmond^A^ et al. (2012)	Frías^B^ et al. (2011)	Frías^C^ et al. (2011)	Morreti^C^ et al. (2016)
SiO_2_	43.01	48.74	55.97	66.61	80.2
Al_2_O_3_	7.0	2.42	12.44	9.46	2.6
Fe_2_O_3_	–	0.47	6.5	10.08	5.6
Fe_3_O_3_	5.23	–	–	–	–
TiO_2_	–	0.16	2.67	2.44	1.4
Ti_3_O_2_	1.56	–	–	–	–
P_2_O_5_	5.82	6.91	0.98	1.04	1.4
CaO	12.75	6.17	0.84	1.43	1.8
MgO	6.70	8.0	0.48	0.92	1.6
Na_2_O	0.20	0.45	–	0.22	0.2
K_2_O	14.14	23.42	0.9	3.19	4.0
SO_3_	1.68	–	1.0	0.10	0.1
SO_4_	–	3.14	–	–	–
MnO	–	0.12	–	–	0.2
MnO_2_	0.53	–	–	–	–
Others	1.35	–	–	–	–
Total Ash	2.93	0.69	–	–	–
Loss of ignition	–	–	17.98	4.27	0.8

^A^Laboratory bagasse ash

^B^Filter bagasse ash obtained from combustion fumes, reaching temperatures ≤ 300 °C

^C^Bottom bagasse ash reaching a boiler temperature ≥ 800 °C

Solving the most difficult ash-related problems include alkali-induced slag, silicate melt-induced slag (ash melt), agglomeration, and corrosion. Some of the most important elements to consider are Si, Al, K, Na, Cl, and S, which are essential for alkaline transformation and alkali-induced slag. Potassium (K) is generally the alkaline metal with the highest concentration in the biomass together with Na, and they are the main cause of problems caused by surface deposits, slagging, bed agglomeration, and corrosion. These metals react easily with silica, even at temperatures below 900 °C, forming silicates or reacting with sulfur to produce alkaline sulfates, which have melting temperatures below 700 °C (Virmond et al. 2012). These sulfates tend to deposit on the walls of the reactor and the heating surfaces by inertial impaction, in addition to adhering to the surface of particles of the bed, provoking the bed's agglomeration, sintering, and defluidization. Agglomeration problems can be defined as the results of two main phenomena: (1) accumulation of salts with a low melting point of potassium and phosphorus, and (2) reaction of potassium phosphate with Si and Ca to generate potassium and calcium silicates of low melting temperature (Niu et al. 2016). Fuel with a low Ca/K ratio results in a high degree of agglomeration, fundamentally when Phosphorus (P) is present, which is another element that forms phosphate compounds that melt at low temperatures. Recent studies on the slag induced by silicate fusion based on the properties of the ash produced in real power plants that use biomass as fuel showed a relationship between the initial deformation temperature (IDT), which is an important parameter to determine the propensity of fuels to form molten or partially molten slag deposits on the heating surfaces of the boilers, and the proportion of silicates present in the ash. An increase in IDT is expected when the content of Al_2_O_3_ and SiO_2_/K_2_O ratio increases, but decreases as K_2_O, SiO_2_, SiO_2_/Al_2_O_3_ ratio, and (SiO_2_ + K_2_O)/Al_2_O_3_ ratio increases (Niu et al. 2016). Silica is undoubtedly the element in greater proportion in the composition of the ashes. Generally, it remains in its crystalline form, not causing problems of slag, fusion, or agglomeration due to its high melting point. However, the silica and the potassium present in high concentrations are directly related to the formation of stubborn surface deposits in the fire tubes and the heating surfaces of the biomass boilers (Niu et al. 2016). Table 9 shows the prediction of the slagging and fouling tendency of the Brazilian sugarcane bagasse ashes through the main correlations used in this field.

**Table 9:** Results of slagging and fouling tendency based on different indexes

Ash Fusibility correlations	Bizzo^A^ et al. (2014)	Virmond^A^ et al. (2012)	Frías^B^ et al. (2011)	Frías^C^ et al. (2011)	Morreti^C^ et al. (2016)
*B/A*_*ratio* + *(P)*_	0.87 High	0.89 High	0.14 Low	0.22 Low	0.17 Low
*Rs*_+ *(P)*_	0.10 Low	0.008 Low	0.01 Low	0.02 Low	0.02 Low
*Fu*	12.5 High	21.1 High	0.15 Low	0.73 Medium	0.73 Medium
*AI (kg/GJ)*	2.3 Slagging certain	1.03 Slagging certain	0.24 Slagging probable	0.75 Slagging certain	0.92 Slagging certain

^A^- Laboratory bagasse ash

^B^-Filter bagasse ash obtained from combustion fumes, reaching temperatures ≤300 °C

^C^-Bottom bagasse ash reaching a boiler temperature ≥ 800 °C

An important conclusion is that there are different behaviors between the ashes obtained in laboratories and industrial ashes obtained from real operating plants. The base-to-acid index (*B/A*_*ratio*_) and fouling index (*F*_*u*_) clearly show a high tendency of laboratory ash to agglomerate, and foul compared to industrial ash. This fact may be fundamentally motivated by important differences in the operating and equipment conditions generated, especially regarding the heating rate and controlled atmosphere, which are quite different between an electric furnace and a real boiler. The chemical analysis of the ashes shows a higher concentration of Al_2_O_3_ in the industrial ashes, which has a positive effect on the IDT, which means a significant increase in the ash fusibility index. On the other hand, laboratory ashes have a higher amount of phosphates (P_2_O_5_) that exhibit eutectic temperatures as low as 590 °C (Niu et al. 2016), which favors problems of melting at low temperatures during combustion. Besides that, research indicates that ashes with SiO_2_ content less than 47 wt.% have higher alkali metal losses. In contrast, ashes with high contents of SiO_2_ tend to retain these elements in the crystalline and melt structures, diminishing their concentration (Niu et al. 2016).

*Rs* index shows incoherent predictions reaffirming that these correlations must be used with great care when applied to biomass combustion since these correlations were obtained for coal combustion ashes. This inconsistency is partly due to the lower levels of sulfur concentrations in biomass compared with coal. The alkali index (*AI*) indicates consistency in the results for all ashes analyzed, predicting a high probability of deposit formation. Although in this study, the composition and fusibility of the ash have not been analyzed experimentally, we can affirm that, in the case of Brazilian sugarcane bagasse, it is expected a high probability of deposit formation with a high tendency towards fouling and slagging that can affect the performance in fluidized bed biomass boilers or gasifiers. Which can cause sintering and agglomeration of the bed, favoring defluidization, according to the studies reported by Bizzo et al. (2014), Virmond et al. (2012), and Fioranelli (2023). Therefore, it is suggested that in the design of fluidized bed plants using bagasse as fuel, adequate ash removal/pretreatment system should be considered, to avoid operational problems such as those previously mentioned. On the other hand, studies show that a high ash content will not necessarily have a negative impact on the thermochemical conversion processes, i.e., in the case of pyrolysis this high ash content can be translated into a higher biochar yield (Najafi et al. 2023).

#### Real density, apparent density, and bulk density of sugarcane bagasse

**Fig. 6:**
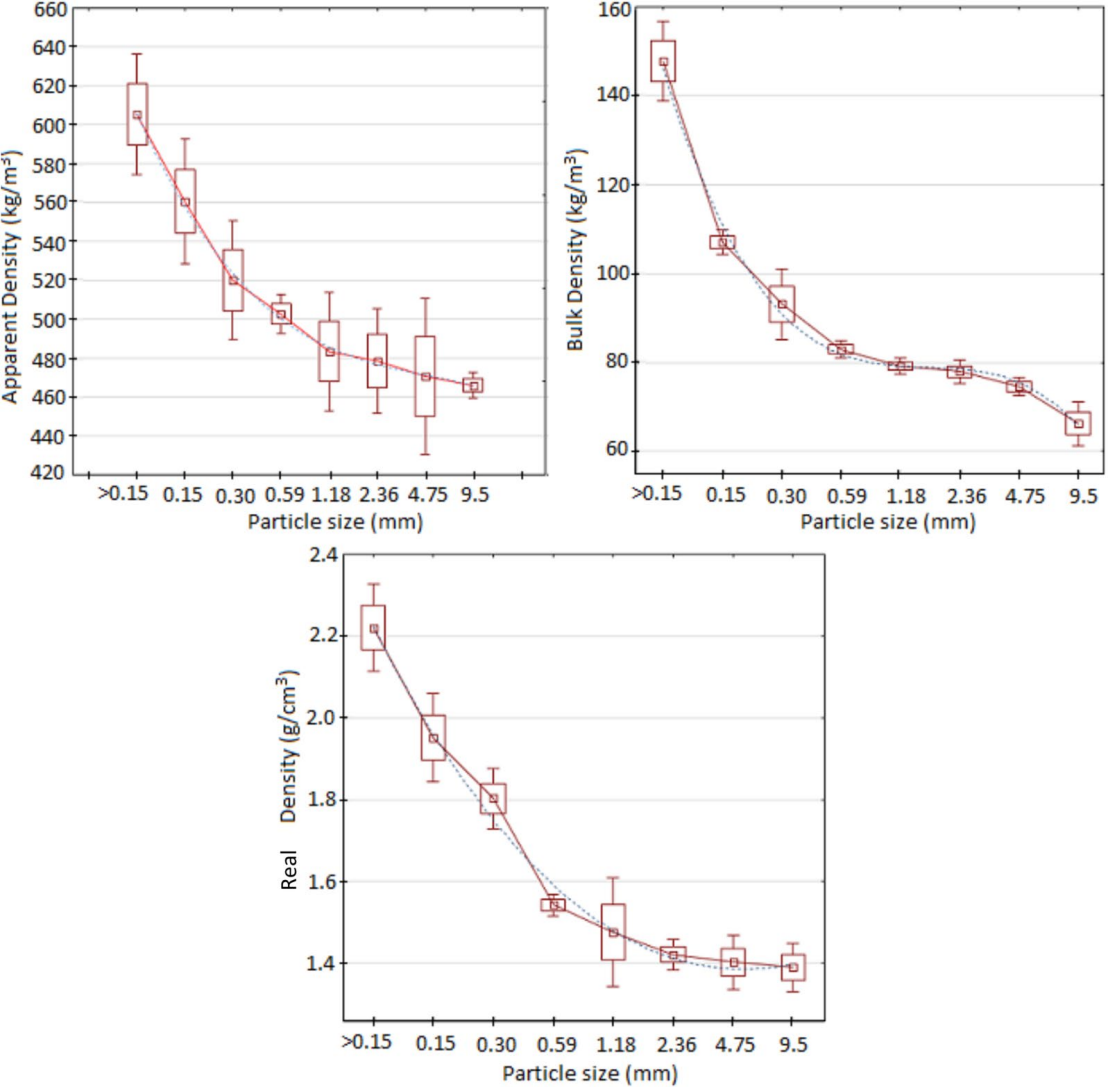
Real, apparent, and bulk densities of sugarcane bagasse particles

Figure 6 shows the values obtained experimentally of the real, bulk, and apparent densities of the sugarcane bagasse particles as a function of the sieve opening used in each size range analyzed. These values represent the mean of three experimental measurements with a 95% confidence interval, indicated by the error bars represented in each graph. In general, the three parameters show a tendency to decrease as the opening of the sieve is greater. This can be translated as the larger the geometric mean diameter of the particles, the lower will be the real, apparent, and bulk density of this type of particle.

For example, the real density increased from 1.39 g/cm^3^ at the sieve opening of 9.5 mm to 2.22 g/cm^3^ for particles retained at the bottom pan of the series of sieves (size < 0.15 mm). For the raw bagasse (geometric mean particle diameter of 0.722 mm), the mean value found experimentally was 1.505 ± 39.2 kg/m^3^ if it is compared to the value obtained by Eq. (9) (1.603 g/cm^3^), which takes into account the density as a function of particle size. It is observed a difference of 6%, meaning that the equation defined above shows a good correlation with experimental results. This value obtained experimentally is very similar to the value reported by Rasul et al. (1999) using a multipycnometer to measure the real density, obtaining a value of 1470 ± 30 kg/m^3^.

The bulk density presents the same tendency of decreasing as the particle size increases for the range of sizes analyzed. The experimental values obtained for this parameter varied from 66.25 ± 2.5 to 147.8 ± 4.6 kg/m^3^ for the smallest particles. In the sieves with a range of size from 0.59 to 4.75 mm, the bulk density remained practically constant with values between 82.98 ± 1.01 and 74.61 ± 1.06 kg/m^3^, respectively. This behavior of the bulk density is due to the bulky nature of the larger particles, which occupy a pore volume greater than the smaller particles so that the smaller particles will have higher bulk densities up to a certain particle diameter. The fact that, in the range of sizes of 0.59–4.75 mm, the bulk density can have a little variation may be due to the characteristics of the particles retained in each screen opening, which are long and thin with a high aspect ratio, which can cause that the volume of pores between the particles to remain practically constant in this range of sizes, independent of their size. The experimentally obtained value for raw sugarcane bagasse was 91.46 ± 1.91 kg/m^3^. This value is approximately 40% lower than the one reported by Conag et al. (2018). Using the reported tapped bulk density as a reference; this difference may result from the conditions under which this parameter was determined in both studies, considering that the degree of compaction directly influences it. According to Rein (2007), in the case of sugarcane bagasse stored in large stacks with determined moisture, the specific value of bulk density may be around 200 kg/m^3^. On the other hand, with the new methods used in the sugar mills for the extraction of the juice and considering the bagasse composed of fine fibers with some degree of compaction, the bulk density can be up to 90 kg/m^3^ (Rein 2007), practically equal to obtained in this work. The value determined analytically (84.96 kg/m^3^) for the bulk density through Eq. (9) was, in this case, underestimated by 7.1% concerning the experimental value.

In the case of the apparent density or particle density, as it is also known, the determination of this parameter using a liquid pycnometer to completely fill the pores present in the particles of bagasse with water is very difficult and laborious, so rigorous criteria should be analyzed to carry out these experiments (Rasul et al. 1999). The trend of this parameter was the same as the one presented previously by the real and bulk densities, increasing as the mean diameter of the particles decreased. It increases from 466 kg/m^3^ at a particle mean diameter of 7.125 mm to 605 kg/m^3^ for the particles retained in the bottom pan. In a comparison made with the study reported by Alarcon et al. (2006), a large difference in the values found can be seen, even for practically equal particle sizes; for example, in particles with a mean diameter of 0.15 mm, the value reported (402.8 kg/m^3^) differs in 28% from the experimental value determined in this work (560.5 kg/m^3^). In other ranges of particle sizes, the differences are still greater. The reason for these large differences in particle density values may lie in the method used to determine this parameter. In the case of the aforementioned study, the determination of this parameter has been made using the Ergun method, which is based on theoretical equations and measurements of the pressure drop caused when a gas flows through a fixed bed of porous particles, very different from the liquid pycnometer technique used in this work. It is valid to point out that the authors call this parameter real density when, in fact, they are referring to apparent particle density or particle density. So, it must pay close attention to the definition presented by each author for each of these parameters.

Nevertheless, Rasul et al. (1999) obtained particle densities of 220 kg/m^3^ for pith, 520 kg/m^3^ for fibers, and 550 kg/m^3^ for skin, which are the main components of sugarcane bagasse, using a liquid pycnometer. Assuming a proportion of pith at about 5%, fibers at 73%, and rind at 22%, found in a typical sample of bagasse, it results in an average value for the particle density of 492 ± 15 kg/m^3^, which shows a good agreement with the value determined experimentally for the bagasse “*in natura*” analyzed in this work (484.63 ± 11.63 kg/m^3^). With Eq. (9), it has been possible to obtain analytically the value of particle density of 505 kg/m^3^, presenting a tendency to overestimate experimental values by approximately 4%. The value obtained is within the expected range for this type of biomass, considering the values reported for this parameter in the studies of Gomez et al. (2012) and Abdullah et al. (2003) for other types of biomasses with similar physical characteristics to sugarcane trash (622 ± 50 kg/m^3^) and palm fiber (407.36 kg/m^3^), respectively. Arithmetic mean particle diameter, real density, particle density, and bulk density of sugarcane bagasse are given in Table 10.

**Table 10:** Main results of experimental densities

Mean diameter (mm)	Real density (kg/m^3^)	Particle density (kg/m^3^)	Bulk density (kg/m^3^)
9.50	1389 ± 30	465.9 ± 3	66.3 ± 2.5
7.125	1402 ± 34	470.6 ± 20	74.5 ± 1.1
3.555	1421 ± 18	478.6 ± 14	77.9 ± 1.3
1.770	1476 ± 68	483.3 ± 15	79.3 ± 0.9
0.885	1542 ± 13	502.6 ± 5	82.9 ± 1.0
0.722^*^	1505 ± 39	484.6 ± 16	91.5 ± 1.9
0.445	1802 ± 37	520.0 ± 15	93.1 ± 4.0
0.225	1952 ± 55	560.5 ± 16	107.1 ± 1.4
0.075	2221 ± 54	605.2 ± 16	147.8 ± 4.6

^*^bagasse "in natura" (composed of all sieve fractions and with geometric mean diameter ${\overline{\text{d}}}_{\text{p}}$)

The densities of the particles in thermochemical conversion processes also greatly influence the final gas conversion and process yield. Smaller particles are expected to have higher combustion rates and ignition front speed. This would be expected behavior in biomasses where larger particles have higher density, lower combustion or devolatilization rates, and consequently lower ignition front speed (Ryu et al. 2006). A different behavior can be expected in the case of bagasse particles, as particles with larger average diameters present lower densities due to higher aspect ratios and lower sphericity. This leads to the appearance of larger interparticle spaces, creating preferential channels that allow the passage of part of the airflow and gasses, causing the ignition front to propagate much faster around these spaces (Ryu et al. 2006).

Studies show that nearly spherical particles react more slowly than less symmetric particles, indicating that even at relatively small particle sizes, sphericity plays an important role in the overall conversion rate (Lu et al. 2010). In the case of bagasse, this should occur in the smaller particles, which have greater sphericity and density, so that the combustion rate should be lower, with a tendency to increase CO and CH_4_ formation. In contrast, H_2_ formation should remain nearly constant as density increases (Bin Yang et al. 2005).

The density of the inert/biomass mixture can also be affected by the height of the static bed, which directly influences the size and frequency of bubbles when the minimum fluidization velocity is exceeded. In shallow beds, a high frequency of small bubbles is expected, which can entrain the less dense particles and accumulate at the top of the bed, causing a segregation phenomenon (Nienow et al. 1978). Already, in the case of deep beds with a high H/D ratio, the frequency of bubbles decreases, causing less segregation. In practical applications, a static bed height between 250 and 800 mm is recommended depending on the type of biomass, height, and diameter of the reactor (Gómez et al. 1995). Considering the economic aspect, the higher the bed height, the bigger the reactor size and the pressure drop that must be overcome, besides the higher the probability of occurrence of the slugging regime due to the growth of the bubbles in their ascent through the bed.

Bagasse particles with higher density have a lower ratio (*d*_*pb*_*/d*_*pin*_) < 3, with a high tendency to segregate in the bed's upper part, which can cause large fluctuations in the reactor temperature profile and chaotic combustion patterns. This behavior may differ from that expected for hard biomass particles, mainly because there is an inversion in the size/density ratio compared to these biomass (Iannello et al. 2023). In the case of bagasse, the larger particles present lower density than the small particles due to the greater number of interparticle voids. When these larger particles are fed into the reactor, it is to be expected the tendency to be dragged to the top of the bed; however, the establishment of the bubble regimen and the appearance of endogenous bubbles under thermal conversion conditions are going to generate a more vigorous motion due to the eruption bubble in the splash zone, which help a better mixing and homogenization of the bed, dragging part of the biomass particles into the dense bed. This behavior can improve thermal conversion due to the higher residence time of the biomass particle in the bed, which provides a good carbon conversion and a gas with a higher calorific value.

In conclusion, in fluidized bed gasification/combustion systems, biomass particles are generally mixed with an inert material to obtain a good mixing and homogenization of the bed, which will allow a better conversion of the gases and improve the final calorific value of the gas. It is expected that when the particle density ratio between the inert bed material and the biomass (*ρ*_*in*_*/ρ*_*b*_) is between 5 and 5.4 with a biomass mass fraction in the order of 2%, this objective will be achieved (Pérez et al. 2017). This is equivalent to saying that bagasse particle sizes between 0.72 and 4.75 mm with a mean diameter ratio biomass/sand (*d*_*pb*_*/d*_*pin*_) in the order of 3–15 would be the most suitable to obtain a constant medium density that allows a high conversion rate.

Table 11 lists the logarithmic relations between the experimental results of the densities and particle sizes of bagasse. For real density, obtaining a model by nonlinear regression methods has been possible, representing the fit to a logarithmic function of the experimental data with a coefficient of multiple determinations (R^2^) value of 0.83. A similar relationship has been obtained for particle density and diameter with an R^2^ value of 0.87. For bulk density, a logarithmic model that relates the bulk density to the mean diameter of bagasse particles has also been developed, with R^2^ values of 0.73.

**Table 11:** Relations between mean particle size and densities of sugarcane bagasse

Densities (kg/m^3^)	Model	Range (mm)	R^2^
Real density	${\rho }_{r}=\text{1592,3696}-\text{134,5167}\cdot \text{ln}({d}_{pi})$	0.075 < *d*_*pi*_ < 9.50	0.83
Particle density	${\rho }_{p}=\text{501,0018}-\text{21,0072}\cdot \text{ln}({d}_{pi})$	0.075 < *d*_*pi*_ < 9.50	0.87
Bulk density	${\rho }_{bulk}=\text{84,5019}-\text{8,4878}\cdot \text{ln}({d}_{pi})$	0.075 < *d*_*pi*_ < 9.50	0.73

The best fit has been selected based on the highest value of the sum of square error and the higher R^2^ of the model. An ANOVA test has also been performed to confirm the fitness of the data for each regression model using the average values of the three parameters analyzed: real density, particle density, and bulk density data. The results obtained have shown in Table 12 that the three regression models were statistically significant (p < 0.05).

**Table 12:** Results of ANOVA test

	Models					
	Real density		Particle density		Bulk density	
	DF^a^	SS^b^	DF	SS	DF	SS
Total	7	664707.500	7	16972.029	7	4743.465
Model	1	568778.352	1	14626.388	1	3637.675
Error	6	95929.148	6	2345.641	6	1105.790
F^c^	35.575		37.413		19.738	
p^d^	0.001		0.001		0.004	

^a^Degrees of Freedom (DF)

^b^Sum of squares (SS)

^c^ANOVA coefficient (F)

^d^Significance (p)

**Fig. 7:**
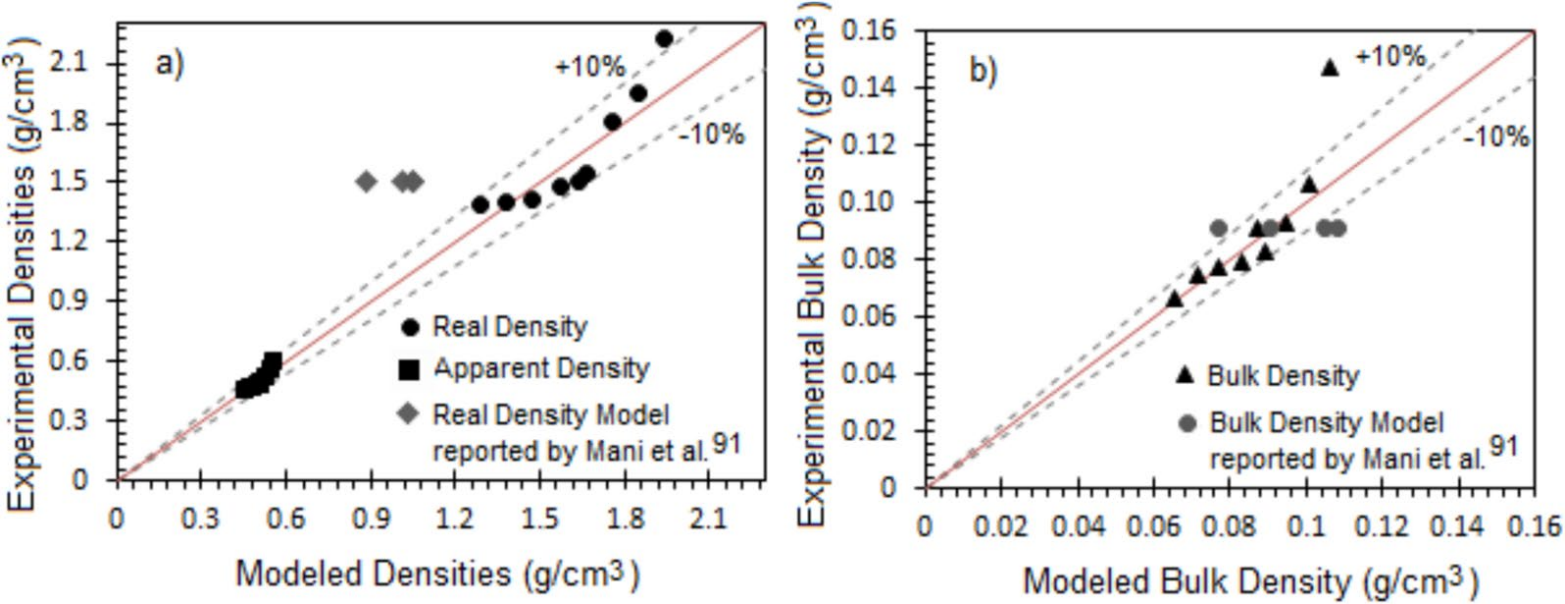
Comparison of experimental and modeled densities for sugarcane bagasse particles in the size range of 0.075–9.50 mm. **a** Real and Apparent densities. **b** Bulk density

Figure 7 shows the behavior of the three models developed for determining the real density and bulk and particle densities, comparing the predicted results with those obtained experimentally for the sugarcane bagasse. Also, the results obtained for predicting raw bagasse's real density and bulk density are presented using other models developed by Mani et al. (2004).

Figure 7a shows the behavior of the new correlations developed in the present work to determine the real and particle densities. The predictive model for the real density presents a good prediction in the range of characteristic mean diameters of 0.075–9.50 mm. The mean relative error reports were between 1.3 and 7.8% of the experimental value for the previous range of diameters. Only particles with sizes lower than 0.15 mm presented the worst prediction, with an average error of 12.6% in relation to the experimental value. Other models developed for other types of biomass were used to predict this parameter, some of them with physical characteristics very similar to those of sugarcane bagasse (Mani et al. 2004). In the case of the raw bagasse with a geometric mean diameter of 0.722 mm, none of the models reported by Mani et al. (2004) adequately predicted the real density, presenting a tendency to underestimate this parameter with errors reported between 30 and 40% of the experimental value. However, the newly developed model predicted this value with an approximation of 92% of the real result for raw sugarcane bagasse. In the case of the particle density model, Fig. 7a shows an excellent correlation with the experimental results. It can be observed that, in the whole range of particle sizes analyzed, the representation tends to coincide with the central line of the figure, which represents the best fit between the modeled value and the experimental result. The relative mean errors reported were between 0.48 and 3.52% in the range of particle sizes analyzed (0.075–9.50 mm), respectively. Similarly to the previous model for determining the real density, higher errors reported corresponded to particles having diameters smaller than 0.15 mm with a mean relative error of 8.23%. In the case of bagasse “*in nature,”* the value of apparent density had an error smaller than 5% of the experimental value (4.78%).

Figure 7b shows the behavior of the bulk density model. The general trend of this model is to acceptably predict the value of this parameter for the entire range of particle sizes analyzed in this work; reporting mean relative errors between 1.3 and 6% of the experimental value. However, for particles with very small diameters (< 0.15 mm), the predicted value was overestimated with a relative mean error of 28% of the experimental result, showing that the model is not very suitable in this class of size. For the case of raw bagasse, a comparison was made with other models reported for biomass particles. Of the four models reported by Mani et al. (2004), only the model developed for wheat straw appropriately predicted this parameter for raw bagasse (geometric mean diameter of 0.722 mm) with an error smaller than 1%. When used in other particle size ranges (${\overline{d}}_{p}=$ 0.225–0.885 mm), the values obtained by the model for wheat straw tended to overestimate this parameter with errors of up to 15%. The other models reported errors in the predicted values between 14 and 70%, respecting the limits of validity established for each one (0.18 < *d* < 1.43 mm and 0.25 < *d* < 1.01 mm) for its correct functioning. When these models are used in other ranges of sizes of bagasse particles (${\overline{d}}_{p}=$ 1.77–9.5 mm), predictions show unrealistic results. Only the model for barley straw was able to adequately predict the bulk density value for particles with a mean diameter of 0.225 mm, with a mean relative error of 2%. For particles with mean diameters of 0.885 mm, the model for wheat straw was the most accurate, predicting the value of bulk density with an error of less than 2%. The foregoing shows that models in the specialized literature developed for specific types of biomasses only adequately predict the bulk density of bagasse particles in very specific sizes, not being suitable in wide ranges of sizes or bagasse particles with a mean diameter greater than 0.885 mm. The newly developed model solves this limitation for the specific case of bagasse by having a considerable range of applications (0.075 ≤ *d* ≤ 9.5 mm). Only for very small particles with a mean diameter smaller than 0.15 mm, the results of the predictions of this model are not adequate, with a tendency to underestimate the real value.

The models were tested for bagasse particles from other regions of the world. It was found that the real density model correctly predicted a theoretical value with relative errors of 3% when compared to the experimental value determined by Rasul et al. (1999). In the case of the particle density model, the predicted value presents a relative error of 8% when compared to the experimental value (Rasul and Rudolph 2000). The bulk density model already underestimated the real value with a relative error higher than 20%. This may have happened precisely because the analyzed particle diameter (*dp* = *0.2 mm*) (Rasul and Rudolph 2000) is very close to the particle diameter value where the model starts to fail, as previously reported (*dp* = *0.15 mm*). It can be concluded that the models are suitable for sugarcane bagasse particles; however, in certain particle sizes, great care must be taken with the prediction, respecting their limitations.

## Conclusion

Characterization of sugarcane bagasse is required to determine its properties and maximize its use in energy production and other applications. The best valorization pathways can be established by identifying its physical, chemical, and thermal properties. Challenges such as seasonal availability, high moisture content, low energy density, and high ash content must be surmounted despite being an attractive renewable feedstock.

Physical characteristics of sugarcane bagasse from various Brazilian cane cultivars were the focus of this study. It examined granulometric composition, showing that real, particle, and bulk densities decrease with increasing particle diameter. These findings align with previous work and form the foundation for developing mathematical models to predict physical behavior. Such knowledge is vital for system design, such as fluidized beds, and selecting materials for gasification or combustion optimization.

However, the study did not extensively explore the effects of varying operational conditions in thermochemical conversion processes, such as different gasification temperatures or pressures, which could influence the performance and efficiency of biomass utilization. Further research could focus on the impacts of operational parameters to enhance energy efficiency and reduce environmental impacts. Additionally, exploring advanced pretreatment methods to improve the quality of sugarcane bagasse for energy conversion and addressing the challenges of ash-related issues in fluidized bed systems could be valuable areas for future investigation.

Advances in processing, handling, and storage, along with supportive policies, are necessary to fully exploit the potential of bagasse. Energetic valorization can reduce waste, avoid environmental impacts, and support a bio-based economy, moving towards a more sustainable energy future.

## Data Availability

The datasets used and/or analyzed during the current study are available from the corresponding author upon reasonable request.

## List of symbols

${d}_{p,i}$: Mean diameter of a particle between two consecutive sieves, *mm*
${\overline{d}}_{p}$: Mean diameter of Sauter, *mm*
*CHNS/O*: Elemental analysis (Carbon, Hydrogen, Nitrogen, Sulfur, and Oxygen), %
*Db*: Burnout index, $\text{\%}/{min}^{4}$
*Di*: Ignition index, $\text{\%}/{min}^{3}$
*HHV*: Higher Heating Value, $MJ/kg$
*S*: Combustion index, $${\text{\%}}^{2}/{min}^{2}*{^\circ C}^{3}$$
$${T}_{burn}$$: Burnout temperature, °C
$${T}_{ign}$$: Ignition temperature, °C
${T}_{peak}$: Peak degradation temperature, °C
*T*_*i*_: Initial temperature, °C
*T*_*onset*_: Process start temperature, °C
*T*_*endset*_: Final process temperature, °C
*T*_*f*_: Final Temperature, °C
${\rho }_{s}$: Density of particles per unit solid volume, excluding pores, $kg/{m}^{3}$
${\rho }_{p}$: Apparent particle density, $kg/{m}^{3}$
$${\rho }_{bulk}$$: Bulk density, $$kg/{m}^{3}$$
